# A *Legionella* effector ADP-ribosyltransferase inactivates glutamate dehydrogenase

**DOI:** 10.1016/j.jbc.2021.100301

**Published:** 2021-01-18

**Authors:** Miles H. Black, Adam Osinski, Gina J. Park, Marcin Gradowski, Kelly A. Servage, Krzysztof Pawłowski, Vincent S. Tagliabracci

**Affiliations:** 1 Department of Molecular Biology, University of Texas Southwestern Medical Center, Dallas, Texas, USA; 2 Institute of Biology, Warsaw University of Life Sciences, Warsaw, Poland; 3 Department of Molecular Biology, University of Texas Southwestern Medical Center, Howard Hughes Medical Institute, Dallas, Texas, USA; 4 Harold C. Simmons Comprehensive Cancer Center, University of Texas Southwestern Medical Center, Dallas, Texas, USA; 5 Hamon Center for Regenerative Science and Medicine, University of Texas Southwestern Medical Center, Dallas, Texas, USA

**Keywords:** ADP-ribosylation, bacterial pathogenesis, bioinformatics, cell metabolism, glutamate, glutamate dehydrogenase, enzyme kinetics, infectious disease, ART, ADP-ribosyltransferase, AYE, ACES-buffered yeast extract, CYE, charcoal yeast extract, GDH, glutamate dehydrogenase, Lart1, *Legionella* ADP-Ribosyltransferase 1, LB, Luria–Bertani, LC-MS/MS, liquid chromatography/mass spectrometry, Lpg, *Legionella pneumophila* gene, mART, mono-ADP-Ribosyltransferase, PDE, phosphodiesterase, PIC, protease inhibitor cocktail, SidE, substrate of icm/dot E, SidJ, substrate of icm/dot J, SDS-PAGE, sodium dodecyl sulfate polyacrylamide gel electrophoresis, T4SS, type IV secretion system, TCA, tricarboxylic acid cycle, WT, wild-type

## Abstract

ADP-ribosyltransferases (ARTs) are a widespread superfamily of enzymes frequently employed in pathogenic strategies of bacteria. *Legionella pneumophila*, the causative agent of a severe form of pneumonia known as Legionnaire’s disease, has acquired over 330 translocated effectors that showcase remarkable biochemical and structural diversity. However, the ART effectors that influence *L. pneumophila* have not been well defined. Here, we took a bioinformatic approach to search the *Legionella* effector repertoire for additional divergent members of the ART superfamily and identified an ART domain in *Legionella pneumophila* gene0181, which we hereafter refer to as *Legionella* ADP-Ribosyltransferase 1 (Lart1) (*Legionella* ART 1). We show that *L. pneumophila* Lart1 targets a specific class of 120-kDa NAD+-dependent glutamate dehydrogenase (GDH) enzymes found in fungi and protists, including many natural hosts of *Legionella*. Lart1 targets a conserved arginine residue in the NAD+-binding pocket of GDH, thereby blocking oxidative deamination of glutamate. Therefore, Lart1 could be the first example of a *Legionella* effector which directly targets a host metabolic enzyme during infection.

Our lab has previously taken a bioinformatic approach to identify atypical and uncharacterized members of the protein kinase superfamily. Several of the outlying kinase families we have characterized are substrates of the *Legionella pneumophila* type IV secretion system (T4SS), including *Legionella pneumophila* gene (Lpg)2603 and substrate of icm/dot J (SidJ) (1, 2). *Legionella* is a gram-negative environmental pathogen and the causative agent of a potentially fatal pneumonia called Legionnaire’s disease. The genus *Legionella*, through horizontal gene transfer from its hosts and cohabiting bacteria, has acquired over 18,000 translocated effectors representing at least 137 different eukaryotic-like domains (3). *L. pneumophila* alone translocates more than 330 effectors, accounting for about 10% of its proteome (4). Because these effectors have evolved to target conserved processes, they represent an orthogonal approach to interrogate eukaryotic biology. Furthermore, effectors are a rich source of structural and biochemical diversity; of the 99 conserved protein domains identified in *L. pneumophila* effectors, 46 are novel (4). Even those effectors with recognizable protein folds sometimes catalyze unexpected reactions (5, 6, 7). For instance, we recently characterized the SidJ effector “pseudokinase,” which adopts a protein kinase fold, but catalyzes polyglutamylation (2, 8, 9, 10). SidJ inactivates the substrate of icm/dot E (SidE) effectors, which harbor phosphodiesterase (PDE) and ADP-ribosyltranferase (ART) domains that cooperate to catalyze phosphoribosyl-linked ubiquitination independent of host E1 and E2 ubiquitin conjugating enzymes (11, 12, 13, 14, 15, 16, 17, 18).

Inspired by the SidE effectors, we sought to identify novel ART folds in the *Legionella* effector repertoire. Members of the ART superfamily transfer ADP-ribose (ADPR) from NAD+, joining the 1′ position of the ribose in N-, S-, or O- linked glycosidic bonds to diverse substrates including proteins, nucleic acids, and small molecules (19). ARTs are widespread in nature, but have extensively diversified in conflict-related systems from bacteria (20), including toxin–antitoxin, virus–host, symbiont–parasite, and antagonistic intraspecific interactions. While poly-ADP-ribosylation is found almost exclusively in multicellular eukaryotes, the prokaryotic ARTs transfer a single ADPR moiety to their substrates (21). Mono-ADP-ribosylation by bacterial toxins, including diphtheria, cholera, pertussis and iota-toxin from *Clostridium perfringens*, is a fundamental pathogenic mechanism for many serious human diseases (22). The protein targets of the most well-known mono-ADP-Ribosyltransferase (mART) toxins include small regulatory G-proteins, actin, and components of eukaryotic translation. While the conventional function of ADP-ribosylation is to lock host proteins in a permanently active or inactive state, the SidE ART domain from *Legionella* reveals that ADP-ribosylation can be an intermediate for other types of unique chemistry. SidE is the only ART domain identified thus far among *Legionella* T4SS effectors. Here we have discovered a mono-ART fold in the *Legionella* effector *Legionella* ADP-Ribosyltransferase 1 (Lart1), which bears only 19% sequence identity with the SidE ART domain. Lart1 was identified as a substrate of the *L. pneumophil*a T4SS in a screen based on translocation of a β-lactamase fusion (23), but its activity and substrates are unknown.

## Results

### Identification of a mono-ADP-ribosyltransferase fold in the Lart1 family of *Legionella* effectors

**Figure 1:**
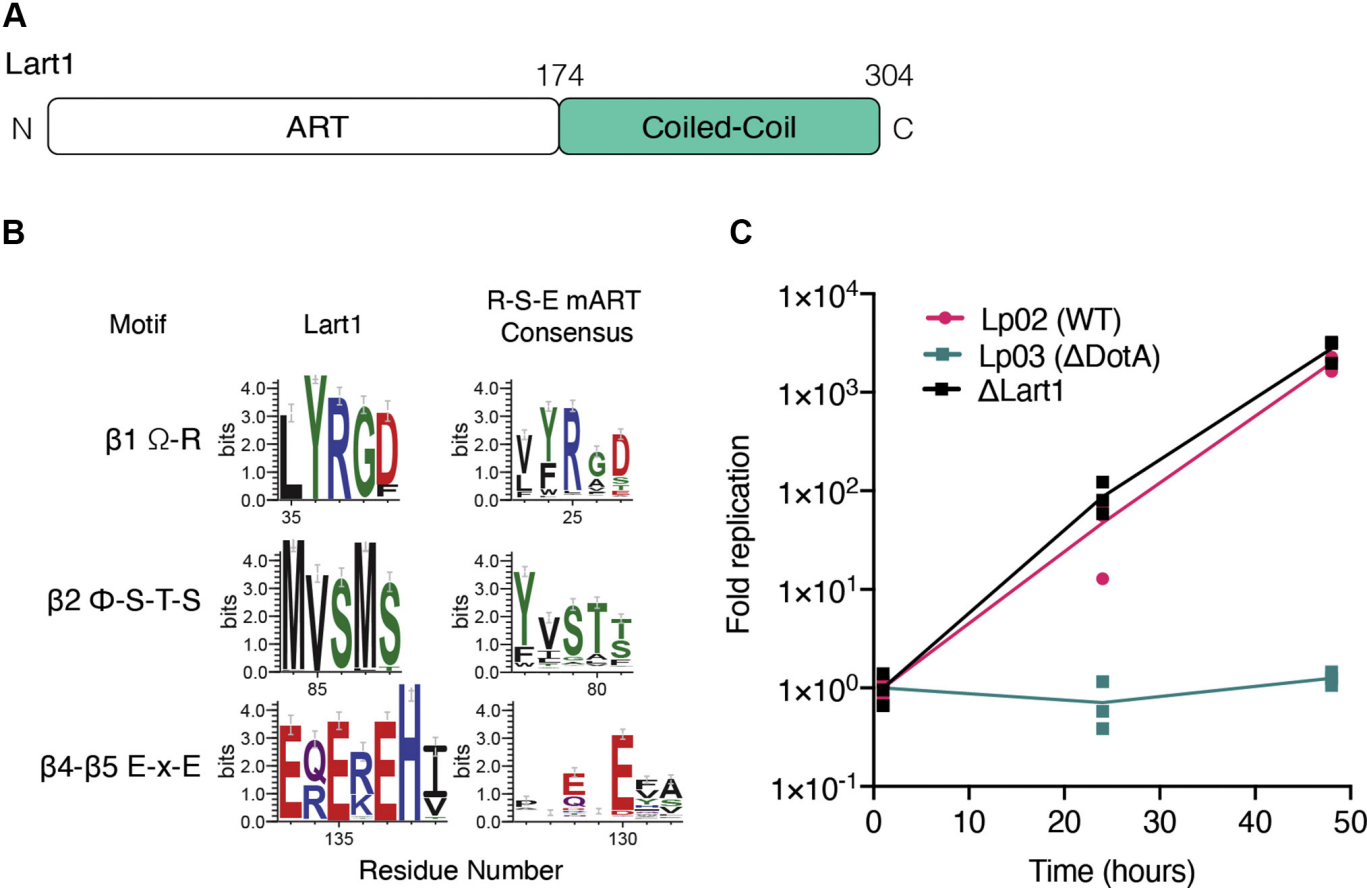
**Lart1 is a mono-ADP-ribosyltransferase.***A*, domain schematic of Lart1. *B*, sequence logos (weblogos) illustrating the conservation of predicted catalytic residues (25) in 81 Lart1 homologs (*left*) and 453 members of the R-S-ExE clade of mARTs (pfam PF01375, enterotoxin a) (*right*). Ω: Aromatic amino acids, Φ: hydrophobic amino acids. *C*, growth curves depicting replication of WT (Lp02), ΔDotA (Lp03), and Lart1 KO (ΔLart1) *Legionella* strains in *A. castellanii* amoeba. Infected amoeba cells were lysed at the indicated time points and bacterial replication was quantified by plating serial dilutions of lysates. Results are obtained from triplicate conditions and are representative of three independent experiments. DotA, defect in organelle trafficking protein; mARTs, mono-ADP-Ribosyltransferases.

We applied a bioinformatic strategy to search for outlying members of the ART superfamily (Fig. S1). Using the FFAS sequence profile algorithm (24), we identified remote similarity to ARTs in the Lart1 family of proteins from the human pathogen *Legionella*. Its closest relatives by FFAS include butterfly DNA ART pierisin, *Salmonella* typhoid toxin, and pertussis toxin with 12 to 16% sequence identity and significant FFAS Z-scores between −10 and −11. The predicted ART domain of the *L. pneumophila* Lart1 lies between residues 1 and 174, while residues 174 to 304 are predicted to adopt a coiled-coil structure (Fig. 1*A*). Sequence alignments identified three conserved active site motifs (R37, ^86^SxS, and ^135^ExE), which place the Lart1 family in the R-S-E clade of mono-ARTs (mARTs) (20, 25) (Fig. 1*B*). Deletion of Lart1 from *L. pneumophila* had no effect on bacterial replication in the amoeba *A. castellani* (Fig. 1*C* and Fig. S2). However, the Lart1 family is widespread in the *Legionella* genus. Eighteen of 48 sequenced *Legionella* species have a Lart1 ortholog (prevalence of 37%), making Lart1 part of the top 100 most common of *Legionella* effector families. Lart1 homologs were also found in three species belonging to the closely related *Fluoribacter* genus, but not in other organisms (Table S1).

### Lart1 ADP-ribosylates yeast glutamate dehydrogenase 2 (Gdh2)

**Figure 2:**
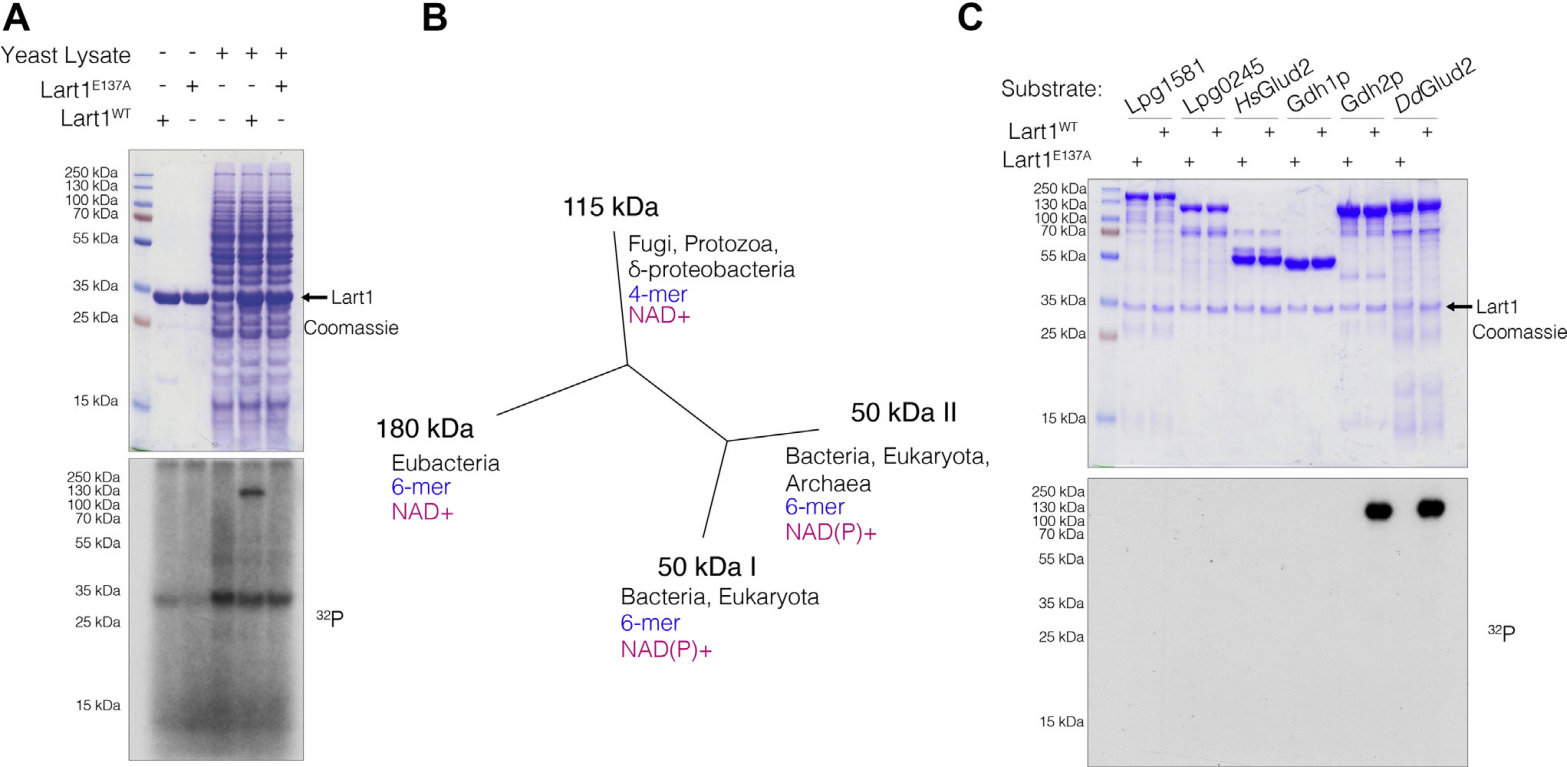
**Lart1 ADP-ribosylates fungal and protozoan isoforms of glutamate dehydrogenase.***A*, incorporation of [^32^P] from [^32^P]-adenylate NAD+ into an unknown 120 kDa protein in a yeast lysate by Lart1, but not the E137A mutant. *B*, dendrogram depicting the four classes of glutamate dehydrogenase isoforms labeled by their subunit molecular weight, adapted from Miñambres *et al.* (26). Each class is annotated with its phylogenetic distribution (*black*), monomer composition (*blue*), and cofactor preference (*magenta*). *C*, incorporation of [^32^P] from [^32^P]-NAD+ by WT Lart1 or the inactive E137A mutant with a panel of glutamate dehydrogenases as substrates: the two *Legionella* glutamate dehydrogenases (Lpg1581 and Lpg0245), human Glud2 (*Hs*Glud2), yeast (Gdh1p and Gdh2p), and *Dictyostelium* Glud2 (*Dd*Glud2). Reaction products were separated by SDS-PAGE and incorporated radioactivity visualized by autoradiography. Lpg, *Legionella pneumophila* gene; SDS-PAGE, sodium dodecyl sulfate polyacrylamide gel electrophoresis.

To identify substrates of this putative *Legionella* ART, we incubated purified recombinant Lart1 with yeast cell extracts and [^32^P]-adenylate NAD+. Lart1, but not the E137A mutant, incorporated ^32^P into a species in a yeast extract that migrated at ∼120 kDa during sodium dodecyl sulfate polyacrylamide gel electrophoresis (SDS-PAGE) (Fig. 2*A*). There were no substrates labeled by Lart1 when mammalian cell lysates were tested (not shown). To identify the ∼120 kDa substrate, we repeated the labeling experiment with biotin-17-NAD+ and used streptavidin resin to enrich biotin-labeled proteins. Bound proteins were eluted by trypsinization and identified by liquid chromatography/mass spectrometry (LC-MS/MS). We prioritized candidate substrates by only considering proteins enriched from reactions containing WT Lart1 but not the E137A mutant. This step eliminated the most abundant proteins identified in both streptavidin pull-downs, including several carboxylase enzymes, which are covalently modified by a biotin prosthetic group. The list of unique proteins was then sorted by MASCOT protein score. The top hit from this sorting was NAD+-dependent glutamate dehydrogenase 2 (Gdh2p, calculated molecular weight of 124.3 kDa) (Table S2).

### Lart1 specifically modifies fungal and protozoan isoforms of glutamate dehydrogenase

GDH enzymes can be broadly divided into four classes (26, 27). The two most common and by far most-studied GDH classes are small (∼50 kDa), hexameric enzymes with widespread phylogenetic distributions. These include the vertebrate GDH enzymes, which are extensively regulated by allosteric feedback (28). A third class of ∼180 kDa, NAD+-dependent GDH is found only in eubacteria. A fourth class of GDH comprises a group of tetrameric, ∼115 kDa enzymes found in fungi, protists, and some deltaproteobacteria (Fig. 2*B*). Yeast Gdh2p belongs to the 115-kDa class, catalyzes glutamate deamination, and confers a growth advantage with glutamate as a sole nitrogen source (29, 30). Although yeast also possess two GDH enzymes of the widespread 50-kDa class (31, 32), neither were identified in our streptavidin pulldowns. Notably, the 115-kDa class is also present in protozoa, the natural hosts of *Legionella*. We selected representative GDH enzymes for heterologous expression in *E. coli* and tested whether recombinant GDH are substrates of Lart1 in reactions containing [^32^P]-NAD+. Lart1 ADP-ribosylated yeast Gdh2p and Glud2 from the amoeba *Dictyostelium discoideum* (*Dd*Glud2) but did not ADP-ribosylate human or *Legionella* GDH homologs, nor did it modify yeast Gdh1p (Fig. 2*C*). Thus, of the substrates we tested, only GDH enzymes of the 115-kDa class are recognized by Lart1.

**Figure 3:**
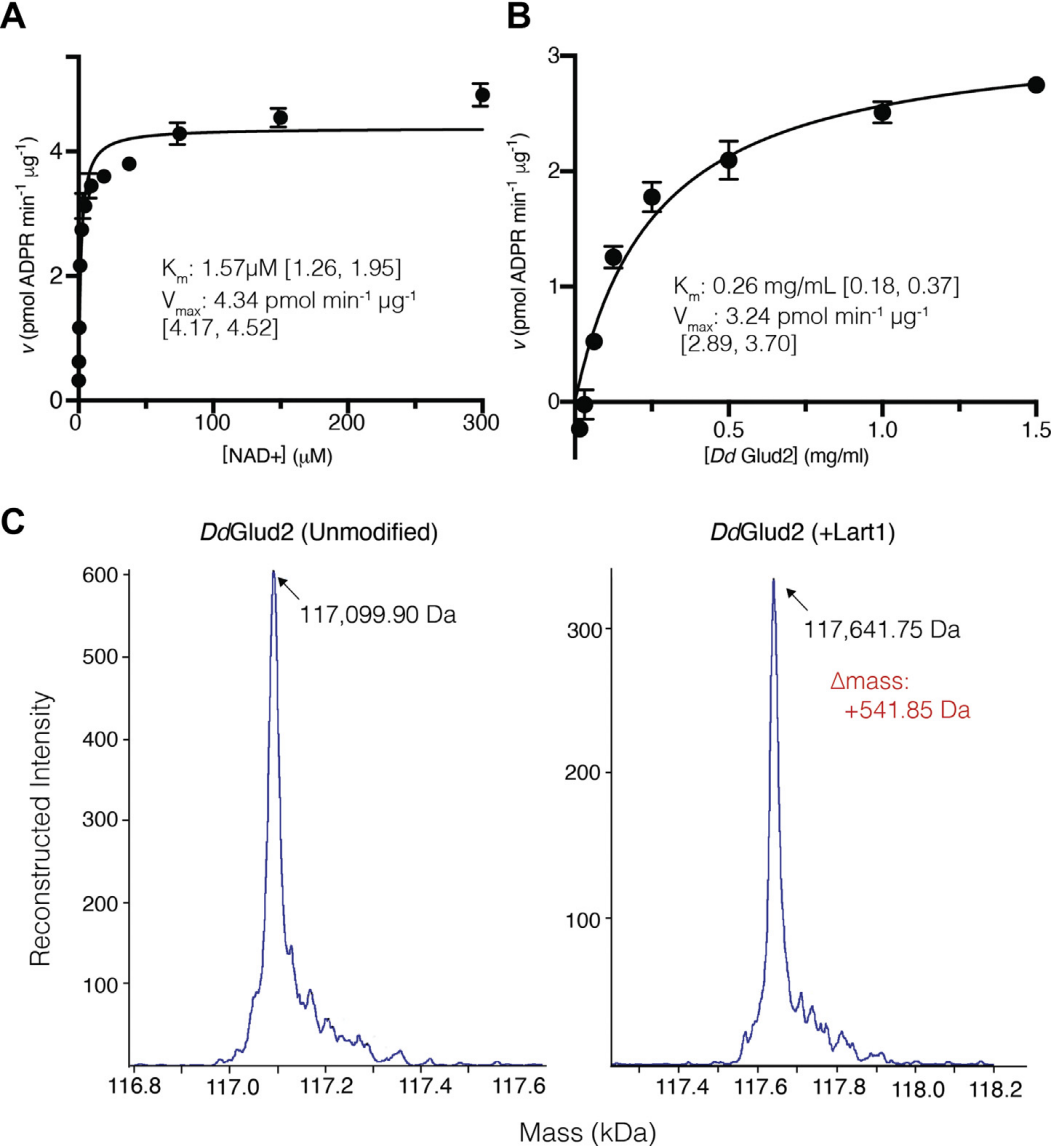
**Kinetic parameters and stoichiometry of the Lart1 transferase reaction.***A*, rate plot depicting the specific activity of WT Lart1 at saturating [*Dd*Glud2] and varying [NAD+]. *B*, specific activity of Lart1 at saturating [NAD+] while varying [*Dd*Glud2]. Km and Vmax (*inset*) are indicated along with a 95% confidence interval. The MW of Lart1 is 34997.6 g/mol. Reactions were performed in triplicate and are representative of three independent experiments. *C*, intact mass spectra of unmodified *Dd*Glud2 (*left*) or after incubation with NAD+ and Lart1 (*right*). The theoretical MW of DdGlud2 is 117142.74 Da and the theoretical mass increase of ADP-ribosylation is 541 Da.

Lart1-dependent ADP ribosylation of *Dd*Glud2 was time-dependent (Fig. S3*A*) and displayed a pH optimum of 6.0 and a slight preference for low (0–50 mM) salt (Fig. S3, *B* and *C*). Like the SidE mARTs (15), Lart1 does not possess NAD+ glycohydrolase activity and only hydrolyzed etheno-NAD+ in the presence of its substrate (Fig. S3, *D* and *E*). Lart1 ADP ribosylation was inhibited by free nicotinamide in ≥ twofold excess of NAD+ (Fig. S3*F*). *Dd*Glud2 was not ADP-ribosylated in reactions without ART enzyme or with ART domains from other *Legionella* effectors, and Lart1-catalyzed incorporation of etheno-ADP-ribose occurred even in the presence of excess unlabeled ADP-ribose (Fig. S4). We determined the kinetic parameters of Lart1 with *Dd*Glud2 as a substrate. The K_m_ for *Dd*Glud2 was 2.22 μM (95% CI 1.54, 3.16) and the K_m_ for NAD+ was 1.57 μM (95% CI 1.26, 1.95) (Fig. 3, *A* and *B*), in accordance with eukaryotic cytosol NAD+ concentrations of ∼0.3 mM (33). Lart1 has a turnover rate (K_cat_) of 0.15 min^−1^ with respect to NAD+ and 0.11 min^−1^ with respect to *Dd*Glud2. The turnover rate and catalytic efficiency of Lart1 are weaker than those of related mART toxins that target proteins, but comparable with members of the DNA-targeting Pierisin family (Table S3) (34, 35, 36, 37). After a 1.5 h reaction, intact mass analysis of *Dd*Glud2 revealed compete stoichiometric addition of a single ADP ribose moiety (+541 Da) (Fig. 3*C*). Lart1 orthologs from four *Legionella* species including *Legionella tucsonensis* (Ltuc1293) *Legionella sainthelensi* (Lsai0094), *Legionella anisa* (Lani2348), and *Legionella longbeachae* (Llo2453) also ADP-ribosylated *Dd*Glud2 in assays using both etheno-NAD+ and biotin-NAD+ as substrates (Fig. S5).

### Lart1 targets a conserved arginine residue in the nucleotide-binding pocket of GDH

**Figure 4:**
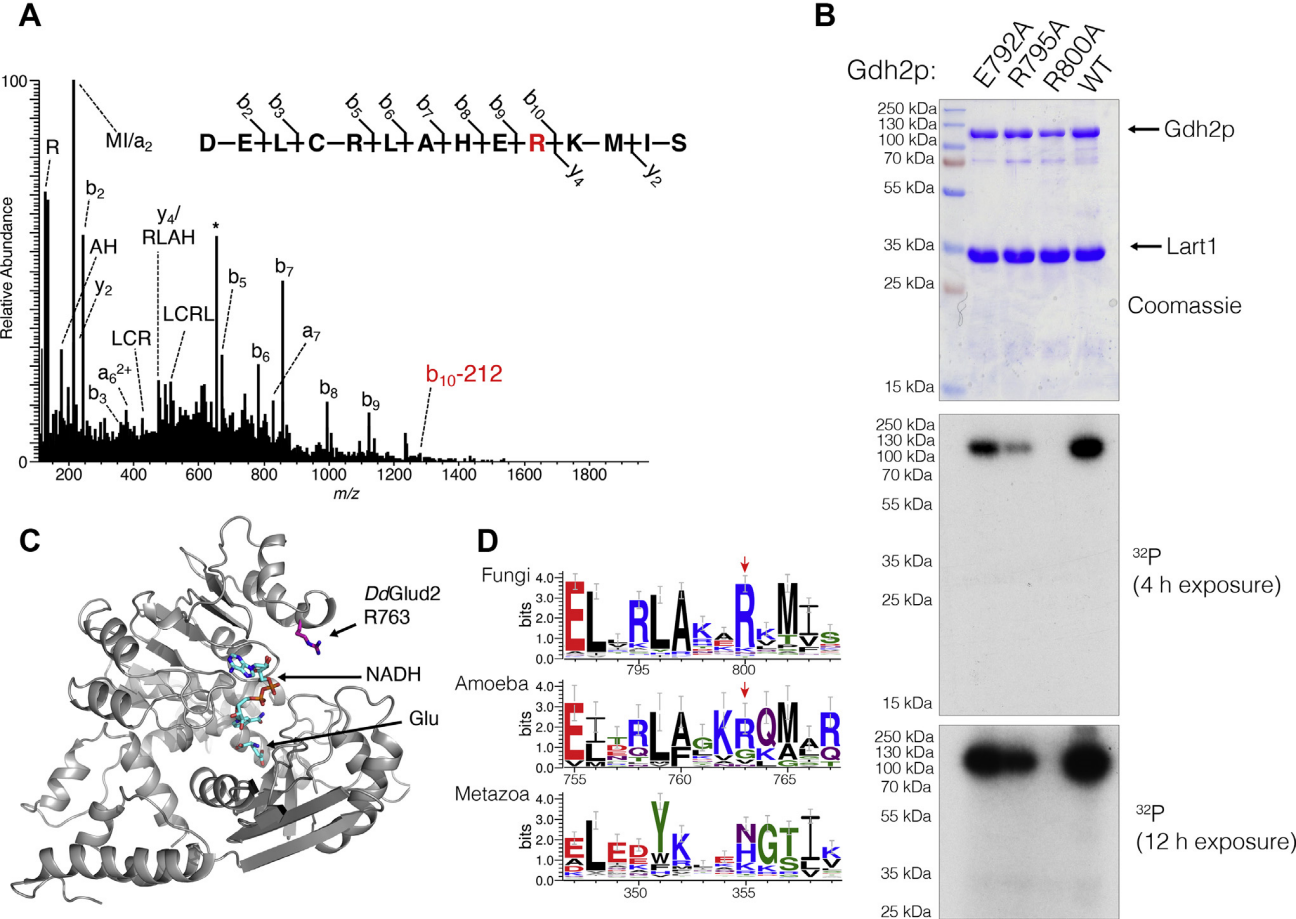
**Lart1 targets a conserved arginine residue in the NAD+-binding pocket of glutamate dehydrogenase.***A*, fragmentation pattern of Gdh2p peptide containing phosphoribosyl-Arg800 identified by LC-MS/MS. The precursor [M+3H]^3+^ ion, *m/z* 657.30, is labeled with an asterisk (∗) and was subjected to HCD fragmentation to generate the spectrum shown. The modification site was localized to arginine 800 highlighted in *red*. The b10 fragment ion containing the modified residue shows neutral loss of the phosphoribosyl group (−212 Da). *B*, endpoint assays depicting incorporation of [^32^P]-NAD+ by WT Lart1 into the indicated alanine mutants of Gdh2p. Mutation of the ADPR acceptor site R800 abolishes ADP-ribosylation. *C*, model of *Dd*Glud2 built by Phyre (64) using *Pyrococcus furiosus* GDH (PDB 1HRD) as a template, indicating the position of the ADP-ribose acceptor residue Arg 763 (rendered in sticks). Bound NADH and glutamate (Glu), (modeled by alignment to liganded bovine GDH, PDB 6DHQ) are rendered in sticks. *D*, sequence logos depicting the conservation of R800 and the surrounding residues in GDH enzymes from fungi (top, based on MAFFT alignment of 125 sequences), amoeba (middle, based on MAFFT alignment of 19 sequences), and metazoans (bottom, based on MAFFT alignment of 145 sequences). *Red arrows* indicate the position of the arginine targeted by Lart1. LC-MS/MS, liquid chromatography/mass spectrometry.

To identify the modified residue of yeast Gdh2p, ADP-ribosylation was enzymatically converted to phosphoribosylation by snake venom phosphodiesterase to aid identification by mass spectrometry (38). LC-MS/MS identified neutral loss of the phosphoribose group in peptides corresponding to Gdh2p 791 to 804 (Fig. 4*A*). Three well-conserved residues in this peptide (E792, R795, and R800) were targeted by alanine mutagenesis, and only the R800 A mutant was not ADP-ribosylated by Lart1 (Fig. 4*B*). Mutation of the corresponding residue (R763) in *Dd*Glud2 also abolished ADP-ribosylation by Lart1 (Fig. S6). There are presently no structures of GDHs of the 115-kDa class. However, the target Arg falls within the relatively well-conserved core nucleotide-binding domain, which can be confidently modeled based on structures of the 50 kDa-class GDHs. A model of *Dd*Glud2 shows that R763 is positioned in a solvent-exposed “lid” within the NAD+-binding pocket, which closes over the ribose moiety when nucleotide is bound (Fig. 4*C*). R763 and the surrounding motif are strongly conserved within fungal and protist GDH enzymes but have diverged in GDH classes from metazoans and bacteria (Fig. 4*D*).

### Characterization of the recombinant NAD+-dependent GDH from *Dictyostelium*

Yeast Gdh2p is a predominately catabolic enzyme that uses NAD+ as a cofactor and catalyzes the oxidative deamination of glutamate (29, 30). Amoeba GDHs are less well characterized and have not been purified to homogeneity, but NAD+-dependent, catabolic GDH activity has been partially purified from the cytosol of *D. discoideum* (39) and *A. castellani* (40, 41). We cloned and purified the 115-kDa class GDH from the soil amoeba *D. discoideum*, which shares 38% sequence identity with yeast Gdh2p. The recombinant enzyme has a subunit M.W. of 117.1 kDa and elutes primarily with mr of 212 kDa (Fig. S7), suggesting a dimer. *Dd*Glud2 reduced NAD+ in the presence of glutamic acid and oxidized NADH in the presence of ammonia and ɑ-KG. Rate plots with varying glutamate and ɑ-KG concentrations are shown in Fig. S8, *A* and *B*. The rate plot for glutamate was roughly hyperbolic and indicates a K_m_ of 18.53 mM (95% CI 10.72, 32.15). ɑ-KG appears to elicit substrate inhibition at concentrations above 50 mM, and the rate plot could not be fit to Michaelis–Menten kinetics. As expected, *Dd*Glud2 did not utilize NADP+ as a cofactor. With substrate concentrations held near K_m_, its rate was not affected by ATP or CTP, but was stimulated 2.5-fold by GTP (Fig. S8*C*).

### GDH is inactivated by ADP-ribosylation

**Figure 5:**
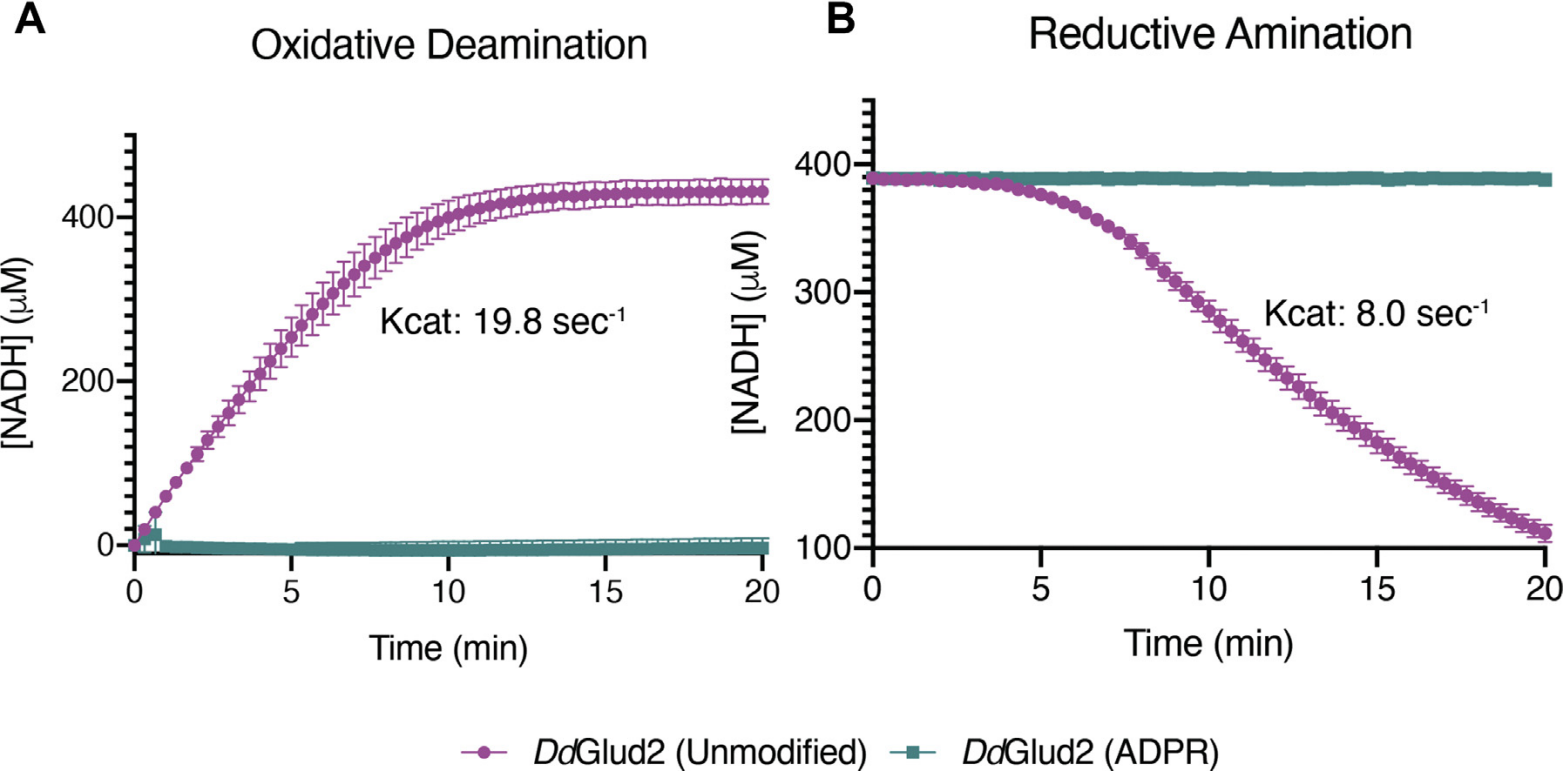
**ADP-ribosylation inactivates *Dd*Glud2**. Reaction progress curves showing (*A*) oxidative deamination of glutamate by unmodified (*magenta*) or ADP-ribosylated (*teal*) *Dd*Glud2, generated by detecting NAD+ reduction to NADH (Abs 340 nm). Reactions contained excess NAD+ and glutamate. *B*, reaction progress curves showing reductive amination by unmodified (magenta) or ADP-ribosylated (teal) *Dd*Glud2. Reactions contained excess ɑ-KG, NADH, and NH_4_^+^. Plots display mean and SD of four replicates from two independent experiments.

ADP-ribosylated *Dd*Glud2 (ADPR-*Dd*Glud2) was prepared in reactions with Lart1 and purified by gel filtration chromatography. In glutamate oxidation assays, ADPR-*Dd*Glud2 was completely inactivated; its catalytic constant was reduced to <1% that of the unmodified enzyme (Fig. 5*A*). Similarly, in reverse reactions with ɑ-KG, ammonia, and NADH, ADPR-*Dd*Glud2 lost detectable activity (Fig. 5*B*). Thus, ADP-ribosylation of host GDH by Lart1 potently inactivates its metabolic function, likely by occluding the NAD+-binding pocket with an ADP-ribose moiety.

## Discussion

We have identified Lart1 as the second *Legionella* effector harboring ART activity, the first being the ART domain in the SidE family of all-in-one ubiquitin ligases. The two types of ART domains are only remotely similar and perform very different functions. This highlights the pathogen’s ingenuity in evolving and repurposing specific enzymatic activities. To our knowledge, Lart1 is also the first *Legionella* effector identified that may target a host metabolic enzyme during infection. Vertebrate GDH is subject to regulation by ADP-ribosylation, presumably as a mechanism to fine-tune insulin secretion (42, 43, 44). However, this ADP-ribosylation is catalyzed by sirtuin 4, which does not adopt an ART fold, occurs on a cysteine residue, and imparts reversible and partial enzyme inhibition (43, 45). In contrast, Lart1 has evolved a distinct mechanism to completely inactivate GDH by targeting a conserved arginine residue in the NAD+-binding pocket.

We have also cloned and purified an amoeba 115-kDa NAD+-dependent GDH, extending observations of crude and partially purified NAD+-dependent GDH activity from several amoeba. While Gdh2p in yeast is thought to be a mitochondrial protein, amoeba GDH activity is present in the cytosol (39, 40, 46). None of our observations indicate that Lart1 would localize to the mitochondria, suggesting that it could encounter amoeba GDH in the cytosol.

Glutamate dehydrogenases are ubiquitous enzymes that occupy a key metabolic branch point, liberating nitrogen from amino acids and supplying carbon chains to the TCA cycle as oxoglutarate. While we were unable to determine if Lart1 can modulate host metabolism during infection, *Legionella* and other intracellular bacteria are entirely dependent on nutrients extracted from their host cell. The concept of “nutritional virulence” postulates that intracellular pathogen virulence strategies are driven by a need to override host nutrient restrictions (47). The levels of free amino acids in host cytosol are insufficient to support the demands of replicating intravacuolar bacteria (48, 49, 50). Thus, secreted effectors probably play a key role in liberating nutrients and triggering host cell processes that increase metabolite levels. For instance, the *Legionella* effector Ankyrin B effector directs widespread K48-linked ubiquitination of host proteins, which are digested by the proteasome. The resulting free amino acids accumulate and supply the dividing bacteria with macronutrients (50). Other intracellular pathogens liberate metabolites by triggering host autophagy (51). Lart1 may also function to increase the concentration of free glutamate by directly inactivating host glutaminolysis. Alternatively, its main action may be through manipulation of α-KG levels. Interestingly, one of the best agar mediums for culturing *Legionella* contains α-KG (52).

GDH may have additional non-metabolic functions targeted through Lart1. Amoeba glutamate dehydrogenases may have important roles in the response to osmotic stress or starvation, cues that induce encystation and restrict bacterial intracellular replication (41, 46, 53). Thus, Lart1 may target amoeba GDH to prevent a conserved stress response.

Deletion of Lart1 had no effect on *Legionella* replication in *A. castellanii*, suggesting that it targets a pathway that is manipulated by additional effectors. Because Lart1 specifically recognizes a subfamily of GDH enzymes only present in fungi and protists, it is unlikely to contribute to virulence in vertebrate macrophages. Lart1 is probably an example of an amoeba-specific “auxilliary” gene that promotes *Legionella* parasitism in its natural hosts and contributes to its broad host range (54).

In conclusion, our results uncover a novel member of the mono-ART superfamily and demonstrate that *Legionella* may have evolved to directly target host metabolic enzymes as part of its pathogenic strategy.

## Experimental procedures

### Reagents

ATP (A2383), CTP, GTP, ɑ-ketoglutaric acid (75890) L-glutamic acid (G1626), NAD+ (10127965001) Etheno-NAD+ (N2630) NADH (10107735001) NADP+ (93205), protease inhibitor cocktail (PIC; 11873580001), and nicotinamide (N3376) were from Sigma. Six-biotin-17-NAD+ (4670-500-01) was from Trevigen (for capture of Lart1 substrate in yeast lysate) and Amsbio (AMS.80610, for assays with Lart1 orthologs). [^32^P]-NAD (NEG023X250UC) was from Perkin Elmer. Tris (2-carboxyethyl) phosphine hydrochloride (TCEP; 20490), PfuTurbo DNA polymerase (50-125-946), and high-capacity streptavidin agarose (20357) were purchased from Thermo Fisher Scientific. High-sensitivity streptavidin HRP (21130) was from Pierce. The ethenoadenosine antibody 1G4 (sc-52666) was from Santa Cruz Biotechnology. Q5 DNA polymerase, 2X Gibson assembly mix, and all restriction enzymes used for cloning were from New England Biolabs.

### Bioinformatic analysis of Lart1

The similarity of Lart1 to known ARTs was established by screening the set of *L. pneumophila* subsp. Philadelphia effectors using the FFAS server (24). Homologs of the Lart1 ART domain and homologs of GDH isoforms were collected using BLAST searches and aligned by MAFFT (55). Sequence logos were produced using the Weblogo 3.0 server (56). The cluster analysis of sequences algorithm (57) was used to represent sequence similarities between ART-like families from the ADP-ribosyl clan (Pfam database identifier CL0084) and four additional novel ART-like families (Lart1, NEURL4, SidE, EspJ). Significant and subsignificant BLAST similarities up to E-value 1 were considered. For the cluster analysis of sequences analysis, ART-like sequence sets were downloaded from the Pfam database reference proteomes rp15 sets (58) or (for the SidE ART domains, bacterial EspJ-like ART domains and human NEURL4-like ART domains) collected using BLAST.

### Generation of plasmids

Lart1, Lart1 orthologs, all GDH homologs, and mutants were cloned into a modified pet28a bacterial expression vector (ppSUMO), containing an N-terminal 6X-His tag followed by the yeast SUMO (SMT3) CDS. The coding sequence of *Dd*Glud2 from the genome of *D. discoidium* AX4 (59) was accessed from dictyBase (60). The *Dd*Glud2 gene (DDB0233691) contains a single intron and a single base (T787), which are removed from the mRNA. The two exons were amplified with NEB Q5 polymerase from *D. discoidium* AX2 genomic DNA and joined by Gibson assembly into ppSUMO. T787 was removed by site-directed mutagenesis. The construct was confirmed by sequencing to be identical to the CDS reported in dictyBase. *Yeast* *GDH1* (*DHE4*, SGD:S000005902) and *GDH2* (*DHE2*, SGD:S000002374) were amplified from BY4741 gDNA, Lart1, Lpg1581, and Lpg0245 (accessed from *L. pneumophila* genome (61) assembly GCA_000008485.1) were amplified from *L. pneumophila* strain Philidelpia-1 gDNA. Llo2453 was amplified from *L. longbeachae* gDNA. The *Hs*Glud2 cDNA clone (NM_012084.3) was obtained from the Ultimate ORF Lite human cDNA collection (Life Technologies). Ltuc1293, Lsai0094, and Lani2348 coding sequences were accessed from NCBI, genome assemblies GCA_001468035.1 (*L. tucsonensis*), GCA_001468105.1 (*L. sainthelensi*), and GCA_001467525.1 (*L. anisa*), as deposited by Burstein *et al.* (4) and were synthesized as gBlocks (Integrated DNA Technologies). Amino acid mutations were introduced *via* Quick Change site-directed mutagenesis as previously described (62). Briefly, primers were designed using the Agilent QuikChange Primer design tool: https://www.genomics.agilent.com and used in PCR reactions to generate the desired mutation using PfuTurbo DNA polymerase. Reaction products were digested with DpnI restriction endonuclease and mutations were confirmed by Sanger sequencing.

### Expression and purification of recombinant Lart1 and Lart1 orthologs

ppSUMO-Lart1 and the E137A mutants were transformed into BL21 Rosetta cells for protein expression. Cells were grown in Luria Bertani (LB) broth supplemented with kanamycin (50 μg/ml) to OD_600_ of 0.8 to 1.1 at 37 °C with constant orbital shaking at 250 rpm. Cells were cooled to 23 °C, and protein expression was induced with 0.4 mM IPTG for 16 to 18 h at 23 °C with orbital shaking at 250 rpm. Cells were harvested by centrifugation at 4000*g* for 15 min and lysed in 50 mM Tris-HCl pH 8, 300 mM NaCl, 1 mM PMSF, 1 mM DTT by sonication. Cell lysates were cleared by centrifugation at 30,000*g* to 35,000*g* for 30 min. The cleared lysate was incubated with washed Ni-NTA beads for a minimum of 1 h at 4 °C. Beads were collected in a gravity-flow column and washed with 20 column volumes of 50 mM Tris-HCl pH 8, 300 mM NaCl, 25 mM imidazole, 1 mM DTT. Proteins were eluted with 50 mM Tris-HCl pH 8, 300 mM NaCl, 300 mM imidazole, 1 mM DTT. Eluates were concentrated to ∼2.5 ml, 6X-His ULP was added, and the protein was transferred to 10,000 Da mwco cellulose dialysis tubing and dialyzed against 5 l of 50 mM Tris-HCl pH 8, 300 mM NaCl, 1 mM DTT overnight at 4 °C with gentle stirring. The cut protein was cleared by centrifugation at 20,000*g* for 10 min and then the volume was increased to 20 ml with fresh dialysis buffer and incubated with fresh Ni-NTA beads for 1 h at 4 °C to bind the cleaved 6X-His-SUMO and 6X-His-ULP. Samples were passed over a second gravity column and the flow-through, containing Lart1, was collected and passed a second time over the Ni/NTA resin. The flow-through was then concentrated and further purified by gel filtration chromatography using a Superdex 75 gel filtration column in with 50 mM Tris-HCl pH 8, 300 mM NaCl, 300 mM imidazole, 1 mM DTT. Peak fractions were collected and concentrated. The purified protein was stored at 2 to 15 mg/ml in gel filtration buffer supplemented with 5% glycerol and flash-frozen in liquid nitrogen prior to storage at −80 °C. Lart1 orthologs from different *Legionella* sp. were purified as above except that ULP treatment, dialysis, and the second Ni/NTA step were omitted for Lani2348 and Llo2453.

### Labeling of Lart1 substrates in yeast lysate

Yeast used in this study was BY4741 [*Mata leu2Δ0 met15Δ0 ura3Δ0 his3Δ1*]. Five ml BY4741 was grown in YPD broth (20 g/l peptone, 10 g/l yeast extract, 2% w/v glucose) for approximately 20 h at 30 °C with orbital shaking at 250 rpm until the culture reached an OD_600_ ≥ 3. The cells were pelleted and washed twice in H_2_O by centrifugation at 800*g* for 5 min. The pellet was resuspended in 500 μl ice-cold IP buffer (50 mM Na-HEPES, 200 mM NaOAc, 1 mM EDTA, 1 mM EGTA, 5 mM MgOAc, 5% w/v glycerol, 0.25% w/v NP-40, 3 mM DTT, 1 mM PMSF, Roche protease inhibitor cocktail (PIC), pH 7.5) and lysed by bead beating (vortexing, 30 s pulses x5 followed by 1 min incubations on ice) with acid-washed glass beads, followed by two subsequent spins at 3000*g* (2 min at 4 °C) and 20,000*g* (10 min at 4 °C). The protein concentration of the cleared lysate was measured by Bradford assay and diluted to 4 mg/ml in IP buffer.

To label substrates in yeast lysate, 20 μl of reactions was prepared with 40 μg yeast lysate or IP buffer, 7.5 μg Lart1 or the E137A mutant, 50 mM Tris HCl pH 6.8, and initiated by adding 100 μM [^32^P]-adenylate NAD+, s.a. 3000 cpm/pmol. Reactions were incubated at 37 °C for 8 min, then stopped by addition of 5 μl 5X SDS-PAGE loading buffer (1× = 12.5 mM Tris-PO_4_ pH 6.8, 10% (w/v) glycerol, 1.25% (w/v) SDS, 0.02% (w/v) bromophenol blue) with β-ME (1% final) and boiled for 10 min. Reactions were resolved by SDS-PAGE, and ^32^P incorporation was detected by autoradiography.

### Streptavidin capture of Lart1 substrate

Biotin labeling was performed in 100 μl of reactions containing 0.3 mg yeast lysate, 0.8 μg Lart1 or the E137A mutant, and 50 μM 6-biotin-17-NAD+ (Trevigen). Reactions were incubated at 37 °C for 15 min, then the volume was increased to 1 ml with ice-cold 1 mM NAD+ in 50 mM Tris HCl pH 7.5. Biotin-labeled proteins were enriched following an adapted BioID protocol (63). Streptavidin agarose resin (Pierce) was washed in 50 mM Tris HCl pH 7.5, and 20 μl resin volume was added to the samples. Samples were nutated at 4 °C for 1 h. The resin was collected by centrifugation (1000*g* × 2 min) and washed twice in 1 ml 2% w/v SDS, then once in buffer 2 (0.1% w/v deoxycholic acid, 1% w/v Triton X-100, 1 mM EDTA, 50 mM HEPES pH 7.5), and once in buffer 3 (0.5% w/v deoxycholic acid, 0.5% w/v NP-40, 1 mM EDTA, 250 mM LiCl, 10 mM Tris HCl pH 7.4). The beads were then collected and suspended in a final volume of 100 μl Tris HCl pH 7.5 and submitted for protein identification by LC-MS/MS.

### Protein identification by LC-MS/MS

An aliquot of streptavidin beads was incubated with trypsin at 37 °C overnight to elute bound proteins. Resulting tryptic peptides were desalted *via* solid-phase extraction (SPE) prior to mass spectrometry analysis. LC-MS/MS experiments were performed on a Thermo Scientific EASY-nLC liquid chromatography system coupled to a Thermo Scientific Orbitrap Fusion Lumos mass spectrometer. Peptides were loaded onto a C18 column (75 μm ID × 50 cm, 2 μm particle size, 100 Å pore size) (Thermo Fisher Scientific) and eluted with a gradient: 0 to 5% B in 5 min, 5 to 30% B in 65 min, 30 to 60% B in 10 min, 60 to 100% B in 8 min. Buffer A consisted of 2% (v/v) acetonitrile and 0.1% formic acid in water. Buffer B consisted of 80% (v/v) acetonitrile, 10% (v/v) trifluoroethanol, and 0.1% formic acid in water. To generate MS/MS spectra, MS1 spectra were first acquired in the Orbitrap mass analyzer (resolution 120,000). Peptide precursor ions were then isolated and fragmented using high-energy collision-induced dissociation (HCD). The resulting MS/MS fragmentation spectra were acquired in the ion trap. MS/MS spectral data was searched using Mascot 2.5 (Matrix Science). Precursor and fragment ion tolerances of 15 ppm and 0.6 Da, respectively, were specified and three missed cleavages were allowed. Oxidation of methionine (+15.995 Da) and carbamidomethylation of cysteine residues (+57.021 Da) were set as variable modifications.

### Purification of *Dd*Glud2

ppSUMO-*Dd*Glud2 was transformed into BL21 Rosetta cells for protein expression. Cells were grown in Luria–Bertani (LB) broth supplemented with kanamycin (50 μg/ml) to OD_600_ of 0.8 to 1.1 at 37 °C with constant orbital shaking at 250 rpm. Cells were cooled to 18 °C and protein expression was induced with 0.4 mM IPTG for 16 to 18 h at 18 °C with orbital shaking at 250 rpm. Cells were harvested by centrifugation at 4000*g* for 15 min and lysed in 50 mM Tris-HCl pH 7.5, 150 mM NaCl, 1 mM PMSF, 1 mM DTT by sonication. Cell lysates were cleared by centrifugation at 30,000*g* to 35,000*g* for 30 min. The cleared lysate was incubated with washed Ni-NTA beads for a minimum of 1 h at 4 °C. Beads were passed over a column and washed with 20 column volumes of 50 mM Tris-HCl pH 7.5, 150 mM NaCl, 25 mM imidazole, 1 mM DTT. Proteins were eluted with 50 mM Tris-HCl pH 7.5, 150 mM NaCl, 300 mM imidazole, 1 mM DTT. Proteins were cut overnight at 4 °C with 6X-His tagged ULP Sumo protease followed by gel filtration chromatography using a Superdex 200 gel filtration column attached to an AKTA Pure FPLC chromatography system (GE Healthcare). Peak fractions were collected and concentrated. The purified protein was stored at 2 to 20 mg/ml in gel filtration buffer protected with 5% glycerol and flash-frozen in liquid nitrogen prior to storage at −80 °C.

### Purification of other GDH homologs

*Hs*Glud2, Lpg1581, Lpg0245 Gdh1p, Gdh2p, and mutants of Gdh2p were purified as described for *Dd*Glud2, except that protein expression was induced at 23 °C, and buffers contained 50 mM Tris-HCl pH 8, 300 mM NaCl, 1 mM PMSF, 1 mM DTT.

### Preparation of ADPR-*Dd*Glud2

ADP-ribosylation was performed in a 100 μl of reaction containing 0.5 mg *Dd*Glud2, 0.1 mg Lart1, 10 mM NAD+, 50 mM Tris HCl pH 8.0, 150 mM NaCl, 1 mM TCEP for 1.5 h at 23 °C. Following a 10 min spin at 10,000*g* to remove aggregated protein, the reaction was cooled to 4 °C and separated on a Superdex 200 increase gel filtration column equilibrated in 50 mM Tris HCl pH 8.0, 150 mM NaCl, 1 mM TCEP. The fractions containing *Dd*Glud2 were collected, concentrated, and submitted for intact mass analysis or used in activity assays.

### Intact mass analysis of ADPR-*Dd*Glud2

Unmodified and ADP-ribosylated *Dd*Glud2 prepared as described above was analyzed using a Sciex X500B Q-ToF mass spectrometer coupled to an Agilent 1290 Infinity II HPLC. Samples were injected onto a POROS R1 reverse-phase column (2.1 × 30 mm, 20 μm particle size, 4000 Å pore size), desalted, and the amount of buffer B was manually increased stepwise until the protein eluted off the column. Buffer A contained 0.1% formic acid in water, and buffer B contained 0.1% formic acid in acetonitrile. The mobile-phase flow rate was 300 μl/min.

The mass spectrometer was controlled by Sciex OS v.1.3 using the following settings: ion source gas 1 15 psi, ion source gas 2 30 psi, curtain gas 35, CAD gas 7, temperature 200 °C, spray voltage 5200 V, declustering potential 80 V, collision energy 15 V. Data was acquired from 1400 to 3600 Da with a 1 s accumulation time and 80 time bins summed. The acquired mass spectra for the proteins of interest were deconvoluted using BioPharmaView v. 2.1 software (Sciex) in order to obtain the molecular weights. The peak threshold was set to ≥5%, reconstruction processing was set to 20 iterations with a signal-to-noise threshold of ≥5 and a resolution of 20,000.

### LC-MS/MS analysis of phospho-ribosylated peptides in *S. cerevisiae* Gdh2

Gdh2p was ADP-ribosylated in 20 μl of reactions with 0.5 mg/ml Gdh2p, 0.125 Lart1, 50 mM Tris HCl pH 7.5, 150 mM NaCl, 1 mM DTT, and 2.5 mM NAD+. After a 20 min incubation at 37 °C, 1.5 μl of 1 M Tris HCl pH 9.0, 0.5 μl of 0.5 M MgCl_2_, and 5 μl of 1 mg/ml snake venom phosphodiesterase I (Sigma) were added to convert ADP-ribosylation to phospho-ribosylation. The reaction was incubated an additional hour at 37 °C and then the reaction was boiled in 1X SDS-PAGE + β-ME sample buffer. The entire sample was resolved by SDS-PAGE. The band corresponding to Gdh2p was excised with a razor and submitted for mass spectrometry.

Samples were reduced with DTT and alkylated with iodoacetamide prior to overnight enzymatic digestion with Asp-N at 37 °C. Resulting peptides were desalted *via* SPE prior to LC-MS/MS analysis. Experiments were performed on a Thermo Scientific EASY-nLC liquid chromatography system coupled to a Thermo Scientific Orbitrap Fusion Lumos mass spectrometer in the same way described above. Samples were searched using Mascot 2.5 (Matrix Science). Precursor and fragment ion tolerances of 15 ppm and 0.6 Da, respectively, were specified and three missed cleavages were allowed. Oxidation (M) (+15.995 Da), carbamidomethylation (C) (+57.021 Da), and phospho-ribosylation (DER) (212.009 Da) were set as variable modifications. MS/MS spectra of phospho-ribosylated peptides identified by Mascot were verified manually.

### ADP-ribosylation assays

Reactions were typically conducted in 20 μl volumes with 50 mM Tris HCl pH 6.8, 50 mM NaCl, 1 mM DTT, contained 0.2 to 0.5 mg/ml GDH substrates, and were initiated with 100 μM [^32^P]-adenylate NAD+, s.a. 500 to 1000 cpm/pmol. Lart1 was added to 0.005 mg/ml for the timecourse, 0.03 mg/ml for the substrate panel, and 0.25 mg/ml for the alanine mutants of Gdh2p. Reactions were conducted at 37 °C for 15 to 20 min or as indicated, then quenched with 2 μl 50 mM unlabeled NAD+ (pH 8), then 5X SDS-PAGE sample buffer + β-ME was added, and the samples were boiled for 10 min. Products were resolved by SDS-PAGE and stained with Coomassie, dried in a gel dryer, and ^32^P incorporation was detected by autoradiography.

To determine the salt and pH optimum for Lart1, 20 μl reactions were performed in 100 mM sodium acetate, 50 mM Bis-Tris, 50 mM Tris-HCl (pH series), and NaCl from 0 to 500 mM. Reactions contained 0.5 mg/ml *Dd*Glud2, 0.007 mg/ml Lart1, and were initiated with 100 μM [^32^P]-adenylate NAD+, s.a. 750 cpm/pmol. Reactions were conducted at 23 °C for 20 min. To determine the Km for NAD+, 20 μl of reactions contained 0.5 mg/ml *Dd*Glud2 with 0.007 mg/ml Lart1 in 50 mM Tris HCl pH 7.5, 150 mM NaCl, 1 mM DTT. Reactions (in triplicate) were initiated with [^32^P]-adenylate NAD+, s.a. 750 cpm/pmol at 13 dilutions from 0 to 300 μM and allowed to proceed at 23 °C for 20 min. To determine the Km for *Dd*Glud2, reactions were performed as above except Lart1 was used at 0.003 mg/ml and [^32^P]-NAD+ was held at 100 μM (s.a. 1000 cpm/pmol) while *Dd*Glud2 was varied from 0.0156 to 1.5 mg/ml. Reactions were stopped with 5 μl of a 5X stop mix containing 80 mM NAD+ in 5X SDS-PAGE sample buffer + β-ME, pH 6.8, boiled 10 min, and resolved by SDS-PAGE. Background was determined in samples that were boiled in stop mix before the addition of [^32^P]-NAD+ mix. Gels were stained with Coomassie blue, washed extensively with water and destain solution to remove background signal, then the *Dd*Glud2 bands were excised with a razor and transferred to scintillation vials. Background radioactivity (typically ∼50 cpm, less than 10% of the lowest experimental value) was subtracted from each measurement. Rate measurements were fit to Michaelis–Menten kinetic models, and Km and V_max_ for substrates were calculated by nonlinear regression using Prism 8.4.1 for macOS (GraphPad Software, www.graphpad.com).

### Etheno-NAD+ hydrolysis assay

Experiments were performed in 384-well Nunc black clear bottom plates in triplicate. Recombinant Lart1 protein or the E137A mutant (0.3 μg/rxn) was incubated with 1, N^6^-etheno-NAD+ (etheno-NAD+) in the presence or absence of *D. discoideum* Glud2 (100 μg/rxn). Enzymes assayed were diluted to 0.06 mg/ml using 50 mM Tris 7.5, 150 mM NaCl, 0.5 mg/ml BSA. In total, 5 μl of enzyme was mixed with 10 μl of substrate or buffer. Reactions were started with 10 μl of etheno-NAD+. Final reaction conditions were as follows: 25 mM Tris 7.5, 90 mM NaCl, 200 μM etheno-NAD+, 1 mM DTT, 0.1 mg/ml BSA in 25 μl volume. ART domain from SdeA (residues 519-1100) +/− Ubiquitin was used as a positive control (0.1 μg SdeA, 50 μg Ubiquitin). Fluorescence at λ_ex/em_ = 310/410 nm was recorded for 1 h using a plate reader. Slopes in the linear part of the assays were calculated using Microsoft Excel 16.43, for each triplicate. Buffer control value was subtracted from obtained V_0_ and plotted using GraphPad Prism 9.

### ADP-ribosylation assays in the presence of nicotinomide

Lart1 was assayed against *Dd*Glud2 in the presence of a 2x dilution series of nicotinamide, in a range of 0.25 to 32× fold over etheno-NAD. These reactions were performed in 20 μl volume at room temperature. In total, 5 μl of Lart1 (0.03 mg/l) was mixed with 5 μl of nicotinamide or buffer, both were diluted in 50 mM Tris 7.5, 100 mM NaCl, 1 mM DTT. Reactions were started by adding 10 μl of master mix solution of aforementioned dilution buffer, containing *Dd*Glud2 and etheno-NAD. Etheno-NAD was added to the master mix shortly before starting the reaction, to avoid its unwanted hydrolysis by *Dd*Glud2. Reactions were stopped after 15 min with 7 μl of 5X SDS-PAGE loading dye containing 20 mM NAD+ (final 5 mM). Final reaction conditions were 50 mM Tris 7.5, 100 mM NaCl, 1 mM DTT, 10 μg *Dd*Glud2 (0.5 mg/ml), 250 μM etheno-NAD+, 0 to 8 mM nicotinamide, 0.15 ug Lart1 (0.0075 mg/ml). Proteins were resolved by SDS-PAGE, transferred to nitrocellulose, then immunoblotting for ethenoadenosine was performed with 1G4 diluted to 1:10,000 in 2% nonfat milk in TBS-T.

### Lart1 ortholog ADP-ribosylation assays with etheno-NAD+ and biotin-NAD+

Lart1 orthologs (Ulp-cleaved or as SUMO-fusions) were assayed against *Dd*Glud2 using either Biotin-NAD+ or etheno-NAD+. In total, 25 μl of reactions contained 0.625 μg of Lart1 ortholog and 5 μg of *Dd*Glud2. Final reaction conditions were 50 mM Bis-tris 6.5, 50 mM NaCl, 1 DTT, with 50 μM Bio-NAD or 250 μM etheno-NAD+. After 30 min incubation at 37 °C, reactions were stopped with 7 μl of 5X SDS-PAGE loading dye. Reactions were separated on 12% SDS-PAGE gel, transferred onto a nitrocellulose membrane, blocked with 5% milk-TBS-T (for ethenoadenosine labeling) or 5% BSA-TBS-T (for biotin labeling). Streptavidin-HRP was diluted 1:40,000 in 5% BSA for detection of biotinylated proteins before washing with TBS-T and detection with chemiluminescence. Immunoblotting for ethenoadenosine was performed as described above.

### ADP-ribosylation assays with other ART enzymes

Reactions were conducted in 25 μl volumes with 50 mM Bis-Tris HCl pH 6.5, 50 mM NaCl, 1 mM DTT, contained 0.2 mg/ml *Dd*Glud2 substrate, 250 μM 1, etheno-NAD+, and were initiated with 0.625 μg Lart1 or the indicated ART enzyme. Some reactions contained 500 μM ADP-ribose where indicated. After a 30 min incubation at 37 °C, reactions were quenched by addition of unlabeled NAD+ to 5 mM and terminated by boiling in 1X SDS-PAGE loading buffer with 1% β-ME.

### Modeling *Dd*Glud2

The entire *Dd*Glud2 protein sequence was submitted for modeling by Phyre (64). In total, 422 residues (40% of the sequence) were modeled with 100% confidence using the highest scoring template (*Pyrococcus furiosus* glutamate dehydrogenase, PDB 1HRD, 22% identity). The binding sites for Glu, NAD+ were determined by superposition of the model with ligand-bound bovine GDH (PDB 6DHQ).

### Glutamate oxidation and ammonia assimilation assays for *Dd*Glud2

Glutamate dehydrogenase activity was measured by continuously monitoring the reduction of NAD+ to NADH spectrophotometrically at 340 nm. Reactions (1 ml volume) contained 5 μg *Dd*Glud2, 100 mM Tris-HCl pH 8.0, 0.1 mg/ml BSA, and 1 mM DTT. NAD+ was held at 5 mM to determine the Km for Glu. NAD+ stock solutions were prepared by dissolving NAD+ (free acid) in H_2_O and adjusting the pH to between 7 and 8 with NaOH, then the concentration was calculated by measuring A_259_ with a molar extinction coefficient of 16,900 l × M^−1^ × cm^−1^ in a quartz cuvette. Glutamic acid was dissolved directly in H_2_O. Reactions were prepared at 9/10 volume without Glu, then aliquoted into clear plastic cuvettes (path length 1 cm). The reaction was initiated by adding Glu (1/10 volume) and pipetting vigorously before initiating measurements. All reactions were performed at 23 °C. Each run was blanked to a cuvette containing reaction mix without enzyme. Under these conditions, the reaction rate was linear between 2 and 5 min and within the sensitivity range of the instrument (A_340_ < 2). [NADH] was determined from A_340_ using the molar extinction coefficient of NADH at 340 nm (6300 l × M^−1^ × cm^−1^). Rate measurements were fit to Michaelis–Menten kinetic models, and Km and V_max_ for substrates were calculated by nonlinear regression using Prism 8.4.1 for macOS (GraphPad Software, www.graphpad.com). ADPR-*Dd*Glud2 was compared with unmodified *Dd*Glud2 in reactions with 5 μg/ml enzyme and saturating substrate concentrations. In some reactions, NADP+ was substituted for NAD+, or ATP, GTP, and CTP were added at 1 mM final with 10 mM Glu and 1 mM NAD+.

To measure ammonium assimilation, ɑ-ketoglutarate (ɑ-KG) stock was prepared by dissolving ɑ-ketoglutaric acid (Sigma) in H_2_O, and fresh NH_4_^+^ stock was prepared by neutralizing NH_4_OH to pH 8 with HCl. Reactions contained 5 μg *Dd*Glud2, 100 mM Tris HCl pH 8.0, 0.1 mg/ml BSA, and 1 mM DTT. NH_4_^+^ was held at 100 mM, and NADH was held at 1 mM while ɑ-KG was varied. The linear portion of each reaction was used to calculate rate plots. ADPR-*Dd*Glud2 was compared with unmodified *Dd*Glud2 in reactions with 100 mM NH_4_^+^ and 40 mM ɑ-KG.

### Generation of ΔLart1 *Legionella* strain

*L. pneumophila* strains Lp02, Lp03 (Lp02 Δ*dotA*), and thymidine auxotrophic derivatives used in this study were derived from *L. pneumophila* Philadelphia-1 strain (65) and were generous gifts from Dr Ralph Isberg. *Legionella* bacteria were maintained on ACES [*N*-(2-acetamido)-2-aminoethanesulfonic acid]-buffered charcoal yeast extract (CYE) agar plates or grown in ACES-buffered yeast extract (AYE) liquid cultures supplemented with ferric nitrate (0.135 g/l) and cysteine (0.4 g/l). Thymidine was added to a final concentration of 100 μg/ml for maintenance of the thymidine auxotrophic strains. Lart1 knockout strains were generated using the R6K suicide vector pSR47s (Kan^R^, *sacB*) (66), a generous gift from Dr Shaeri Mukherjee, UCSF. Briefly, ∼800 bp regions flanking the Lart1 ORF (Fig. S7) were amplified and cloned using Gibson assembly into pET-21a(+), then subcloned into pSR47s to generate pSR47s-Δ*Lart1*, which was maintained in S17-1 λpir *E. coli*. pSR47s-Δ*Lart1* was introduced by electroporation into strain Lp02, and colonies having undergone homologous recombination were selected with kanamycin (20 μg/ml). Merodiploids were resolved on 10% sucrose, and the resulting colonies were screened for loss of Lart1 by PCR and protein immunoblotting. Lart1 complementing strains were generated using the RSF1010 cloning vector pJB908 (Amp^R^ *td*Δ*i*) (67), a generous gift from Dr Ralph Isberg. Transformants were selected on CYE medium without thymidine, and complementation was verified by PCR and protein immunoblotting.

### Production of Lart1 antibodies

Untagged WT Lart1 was purified as described above and used to inoculate rabbits for generation of rabbit antiserum (Cocalico Biologicals). Total IgG was partially purified by ammonium sulfate precipitation (68), and the α-Lart1 antibody was affinity-purified by coupling recombinant Lart1 to a HiTrap NHS-activated HP column essentially as described (69). Antibodies were concentrated, aliquoted, and stored at −20 °C until use at a 1:2000 dilution in 2% nonfat-milk TBST.

### Intracellular replication in amoeba

*Acanthamoeba castellanii* was maintained as a monolayer culture in PYG medium (20 g/l protease peptone, 1 g/l yeast extract, 150 mM glucose, 4 mM Mg_2_SO_4_, 0.4 mM CaCl_2_, 0.1% (w/v) sodium citrate dihydrate, 0.05 mM Fe(NH_4_)_2_ (SO_4_)_2_ × 6H_2_O, 2.5 mM NaH_2_PO_3_, 2.5 mM K_2_HPO_3_ pH 6.5) in tissue culture flasks at 23 °C. Eighteen hours prior to infection, confluent amoeba monolayers were collected by pipetting in ice-cold PBS, resuspended in fresh PYG, counted, and 6 × 10^5^ cells were seeded into individual wells of 24-well plates. One hour prior to infection, amoeba were carefully washed twice, the medium was replaced with *A. castellanii* buffer (70) (4 mM magnesium sulfate, 0.4 mM CaCl_2_, 0.1% (w/v) sodium citrate dihydrate, 0.05 mM Fe(NH_4_)_2_ (SO_4_)_2_ × 6H_2_O, 2.5 mM NaH_2_PO_3_, 2.5 mM K_2_HPO_3_ pH 6.5), and the plates were equilibrated at 37 °C. All subsequent incubations were performed at 37 °C. *Legionella* cultures at postexponential phase were diluted in *A. castellanii* buffer, and ∼6 × 10^4^ bacteria were added to each well for a multiplicity of infection (MOI) of 0.1. Infections were synchronized by centrifugation at 880*g* for 5 min. Infections were allowed to proceed for 1 h, then extracellular bacteria were removed by washing each well three times in *A. castellanii* buffer before adding *A. castellanii* buffer to a final volume of 0.5 ml/well. At timepoints 1 h, 24 h, and 48 h, infected amoeba cells were lysed in 0.05% saponin in H_2_O. Serial dilutions of the infectious inoculum and the amoeba lysate were plated on CYE plates to confirm the MOI and assess bacterial growth.

## Data availability

The authors indicate that all of the data is contained within the article.

## Conflict of interest

The authors declare that they have no conflicts of interest with the contents of this article.
