# Supplementary material for: A Legionella effector ADP-ribosyltransferase inactivates glutamate dehydrogenase
Source: J Biol Chem. 2021 Jan 18;296:100301. doi: 10.1016/j.jbc.2021.100301 (PMC7949102; doi:10.1016/j.jbc.2021.100301)
Supplement: Supplemental Figures and Tables [file mmc1.pdf]

Supporting Information For:

A *Legionella* effector ADP-ribosyltransferase inactivates glutamate dehydrogenase

Miles H. Black[1], Adam Osinski[1], Gina J. Park[1], Marcin Gradowski[2], Kelly Servage[1,3], Krzysztof Pawłowski[2], and Vincent S. Tagliabracci[1,4,5*]

Figure S1

Figure S2

Figure S3

Figure S4

Figure S5

Figure S6

Figure S7

Figure S8

Table S1

Table S2

Table S3

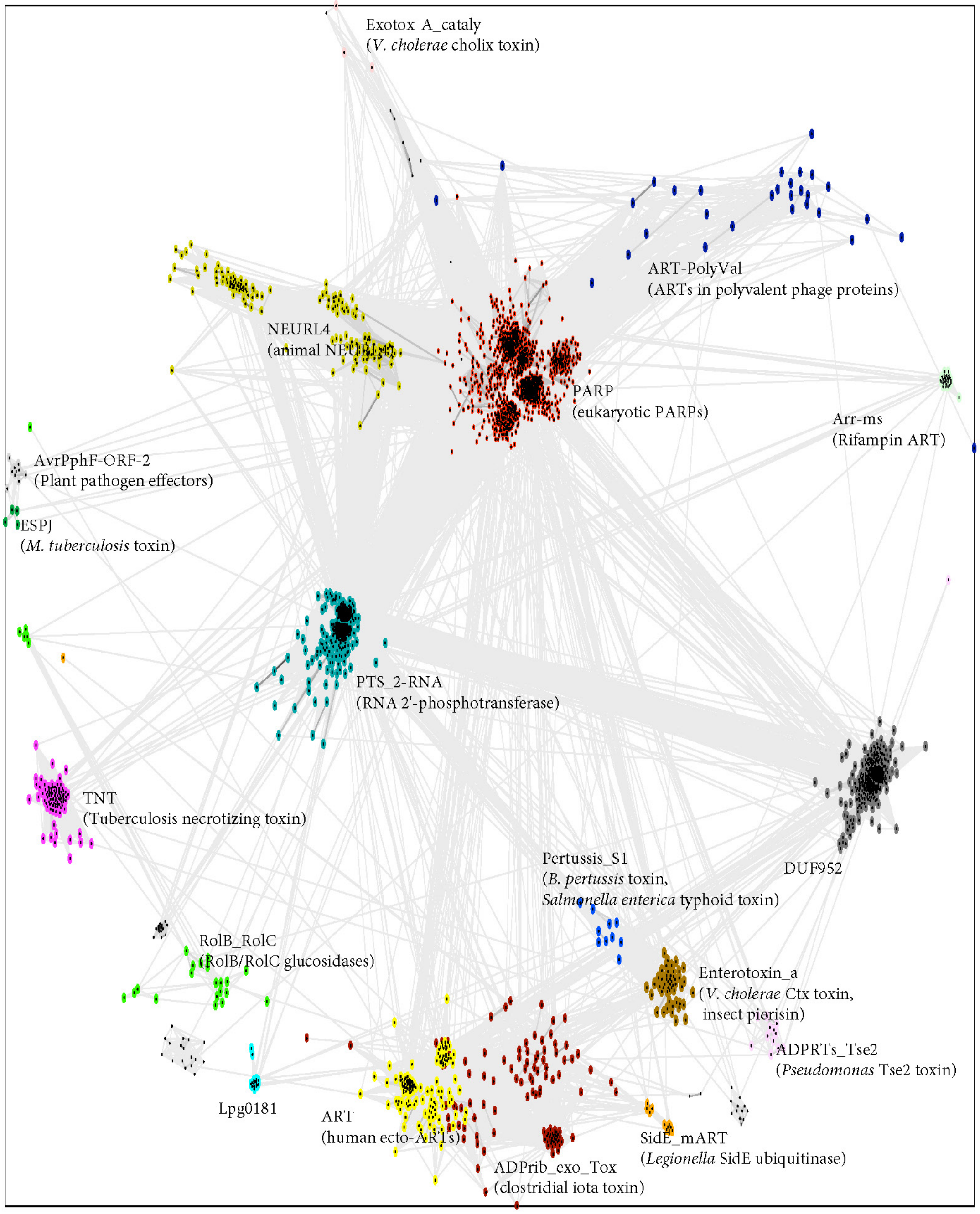

Figure S1. CLANS graph showing sequence similarity-based clustering of ART domain families. The graph shows 2787 representative proteins represented as points; families are colored and annotated by Pfam database identifiers, and examples of family members shown in parentheses. The Lpg0181 (Lart1), NEURL4 and EspJ ART-like families are not defined in the Pfam database and were collected manually.

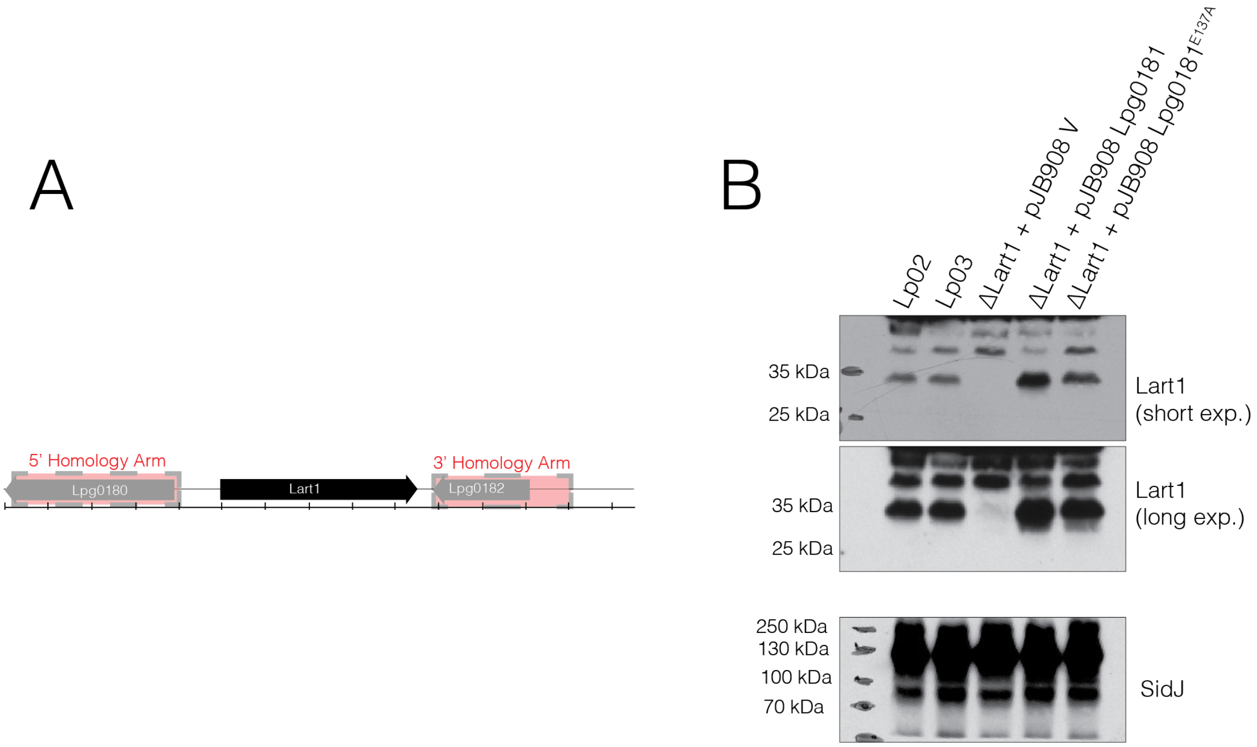

Figure S2. Generation of ΔLart1 *Legionella* strains. (A) schematic of the Lart1 ORF and the position of the homology arms used to generate the knockout strain. Ticks on the scale bar = 200bp. **(B)** Immunoblotting of *Legionella* bacterial pellets harvested from post-exponential liquid cultures and boiled directly in SDS-PAGE sample buffer. The predicted MW of Lart1 is 34.9 kDa.

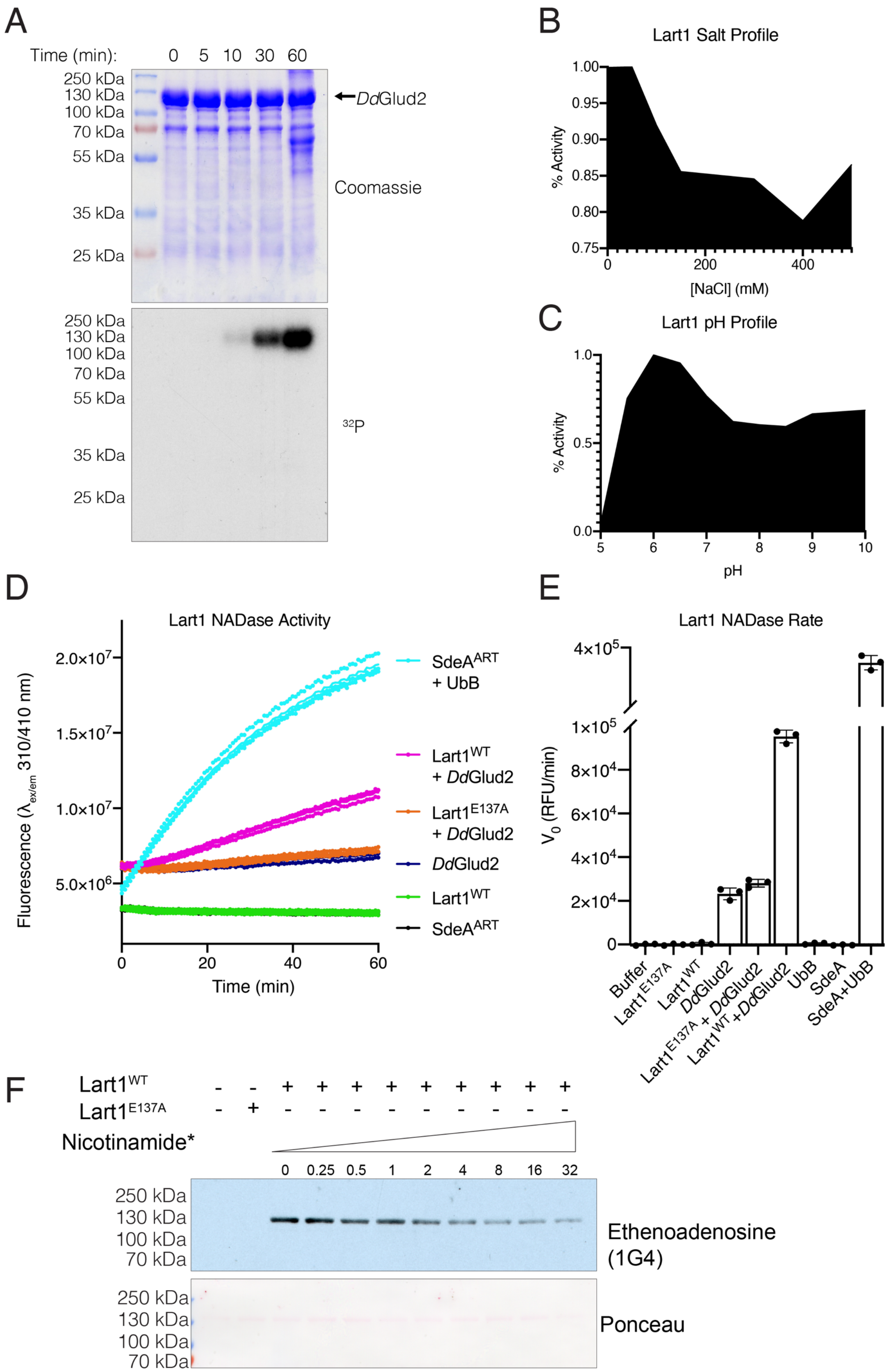

*Fold excess over etheno-NAD+

Figure S3. Characteristics of Lart1 ADP-Ribosylation. (A) Timecourse *in vitro* ART assay showing incorporation of ^{32}P from [^{32}P]-adenylate NAD+ into *Dd*Glud2 by Lart1^{WT}. products were resolved by SDS-PAGE (upper) and visualized by autoradiography (lower). **(B)** incorporation of [^{32}P]-ADPR into *Dd*Glud2 by Lart1^{WT} with varying concentrations of NaCl or **(C)**, buffered at the indicated pH. Reactions were resolved by SDS-PAGE and stained with Coomassie, then radioactive gel bands were excised for scintillation counting. **(D)** Progress curves measuring fluorescent etheno-ADPR release from etheno-NAD+ by Lart1^{WT} and Lart1^{E137A} in the presence or absence of *Dd*Glud2. *Dd*Glud2 alone and the SdeA mART domain (SdeA^{ART}) alone and with its substrate (UbB) were included as controls. **(E)** Reaction rates from **(E)** plotted in a bar graph. **(F)** Lart1-catalyzed ADP-ribosylation of *Dd*Glud2 in the presence of nicotinamide, Etheno-NAD+ was used in these experiments and nicotinamide was added at the indicated fold excess to etheno-NAD+.

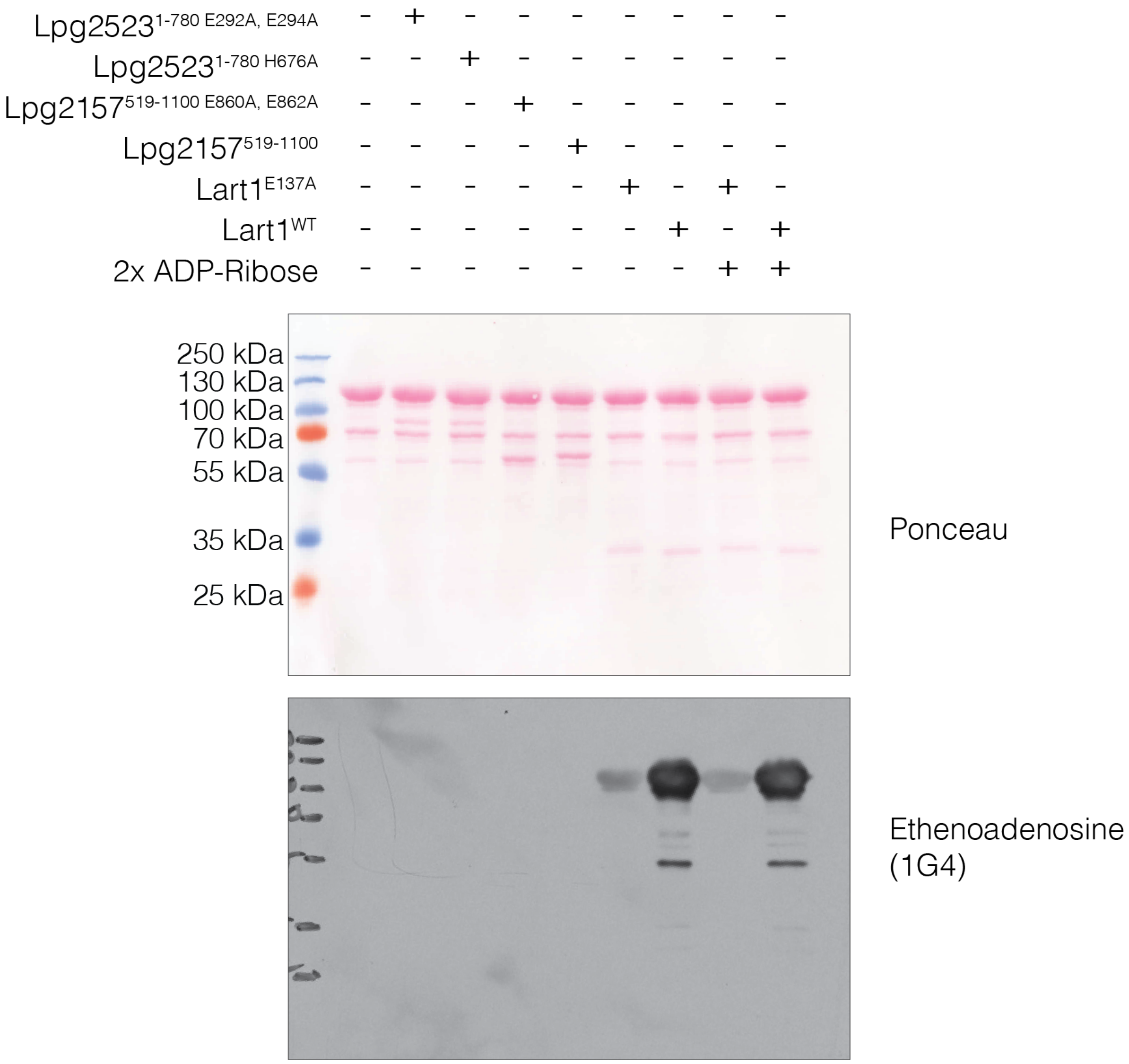

Figure S4. ADP-ribosylation of *Dd*Glud2 is enzyme-catalyzed. ADP-ribosylation reactions with *Dd*Glud2 as substrate were performed with nicotinamide 1,N6-ethenoadenine dinucleotide (etheno-NAD+) with no ART enzyme (lane 1), the Lpg2526 ART domain-containing effector Lpg2523 H676A (lane 2) or its ART catalytic mutant (lane 4), the SdeA (Lpg2157) ART domain and its catalytic mutant (lanes 5 and 6), or WT Lart1 and the catalytic mutant (lanes 7 and 8). In lanes 9 and 10, unlabeled ADP-ribose was added at two-fold excess to etheno-NAD+ as a test for non-enzymatic glycation. Reaction products were separated by SDS-PAGE, transferred to nitrocellulose and visualized by Ponceau staining, then etheno-ADP-ribose incorporation was detected by immunoblotting with the ethenoadenosine antibody 1G4.

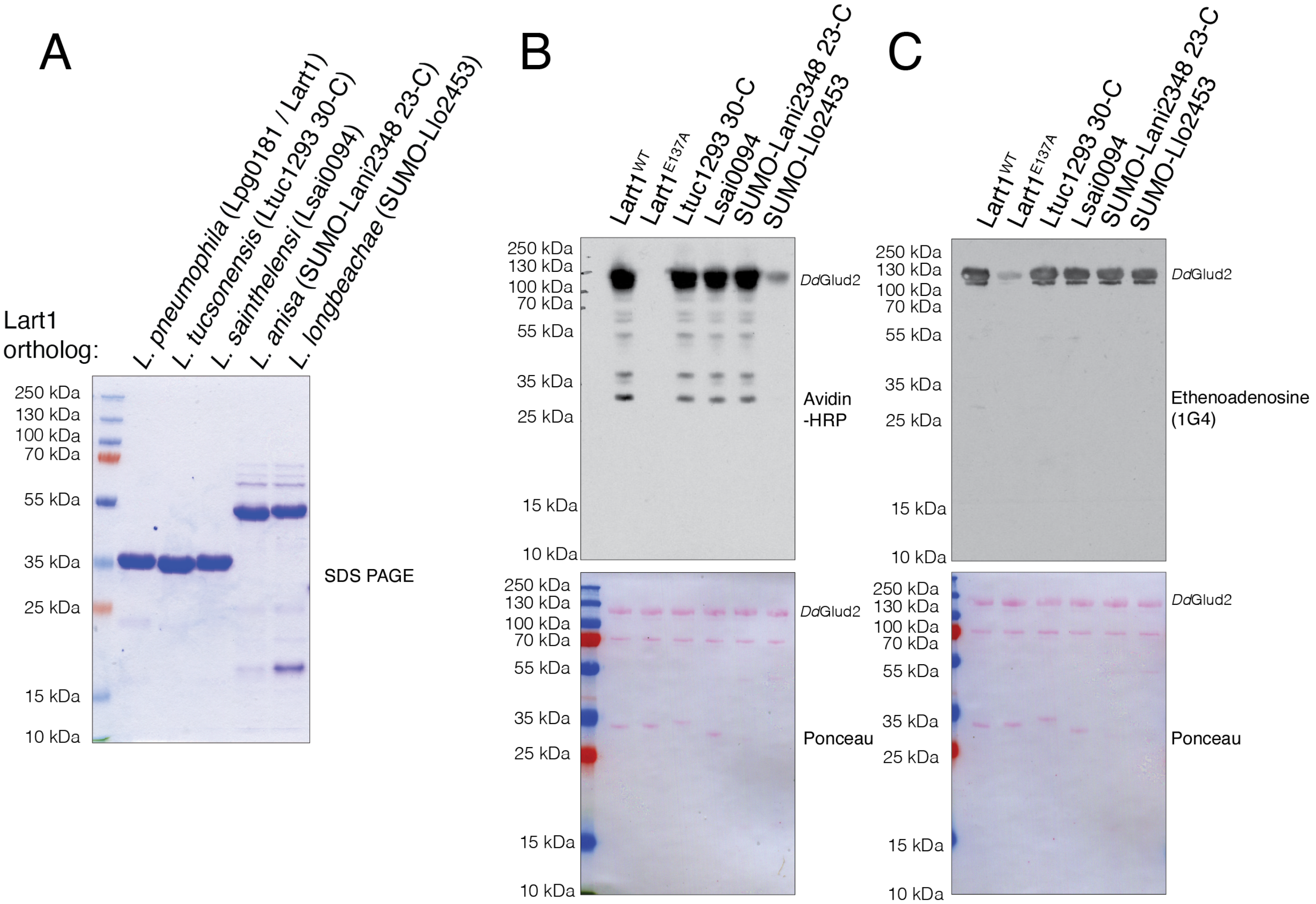

Figure S5. ADP-ribosylation of *Dd*Glud2 by Lart1 orthologs. (A) SDS-PAGE of Lart1 and four orthologs from the indicated *Legionella* species. Lani2348 and Llo2453 were purified as SUMO-fusions and the indicated amino-terminal truncations of Ltuc1293 and Lani2348 were made to improve solubility. **(B)** Incorporation of biotin-ADPR from biotin-NAD+ into *Dd*Glud2 by WT Lart1, the E137A mutant, and the indicated Lart1 orthologs. The reaction products were resolved, transferred to nitrocellulose, and biotin-ADPR incorporation was detected by immunoblotting with HRP- avidin. **(C)** ADP-ribosylation of DdGlud2 as in (B) except that etheno-NAD+ was used as a co-substrate. Etheno-ADPR incorporation was detected by immunoblotting with the anti-etheno 1G4 antibody.

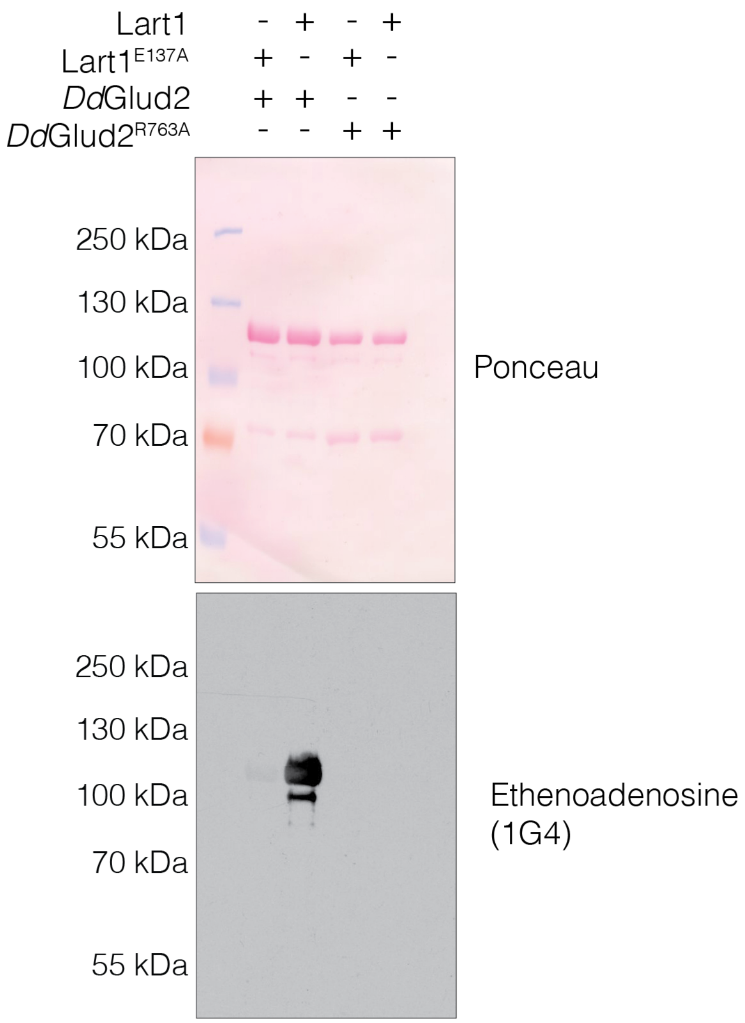

Figure S6. Lart1 targets R763 of *Dd*Glud2. ADP-ribosylation reactions with *Dd*Glud2 or *Dd*Glud2^{R763A} as substrates were performed with etheno-NAD+ and Lart1 or the E137A mutant. Reaction products were separated by SDS-PAGE, transferred to nitrocellulose and visualized by Ponceau staining, then etheno-ADP-ribose incorporation was detected by immunoblotting with the ethenoadenosine antibody 1G4.

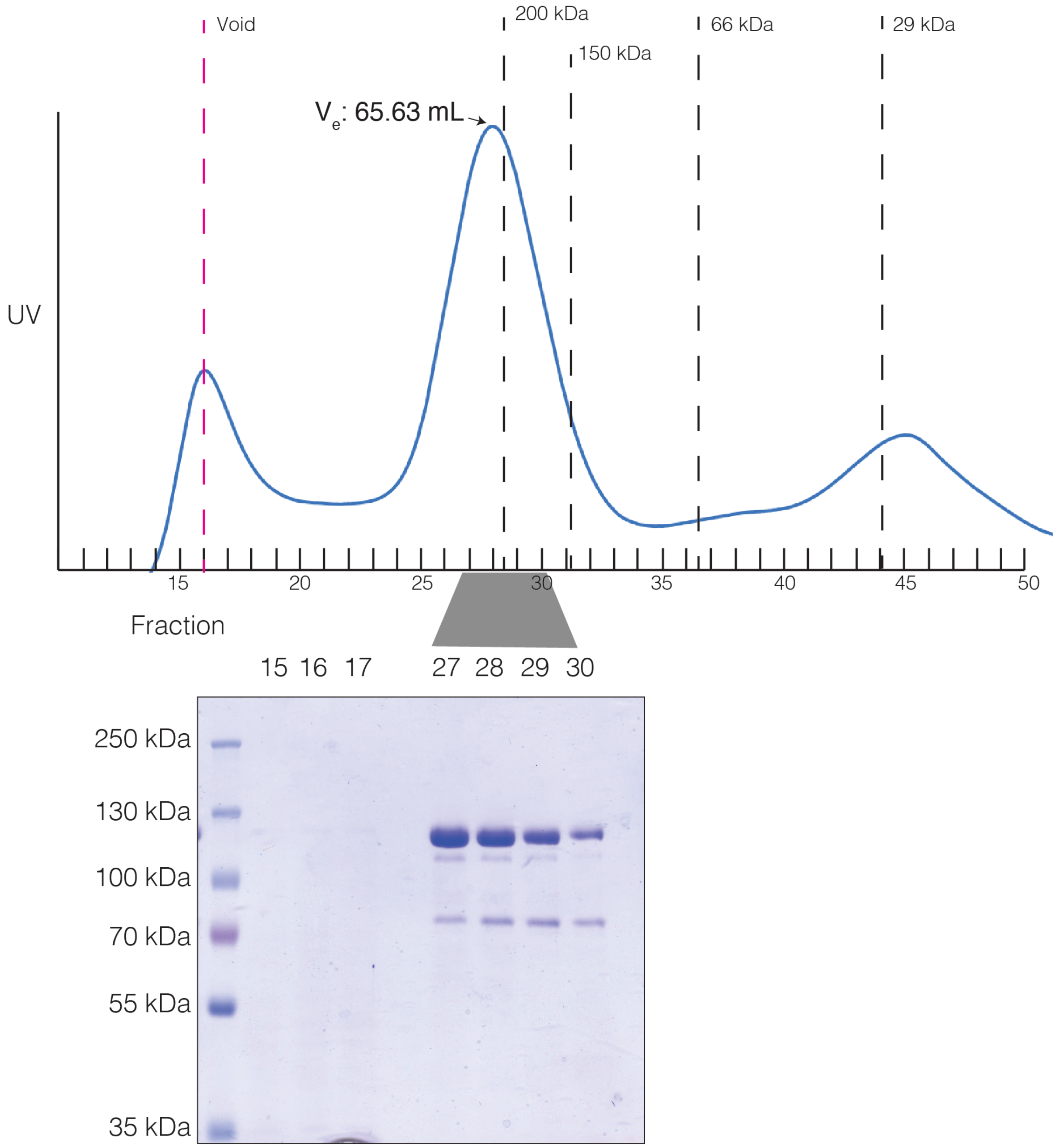

Figure S7. Size-exclusion chromatography of *Dd*Glud2. Recombinant *Dd*Glud2 was subjected to chromatography on a Superdex 200 column (GE). The UV trace (above) is superimposed on peak elution volumes of molecular weight standards (dotted black lines) and void elution volume (blue dextran, red dotted line). Fractions corresponding the void and peak elution were separated by SDS-PAGE and visualized by Coomassie staining (lower panel).

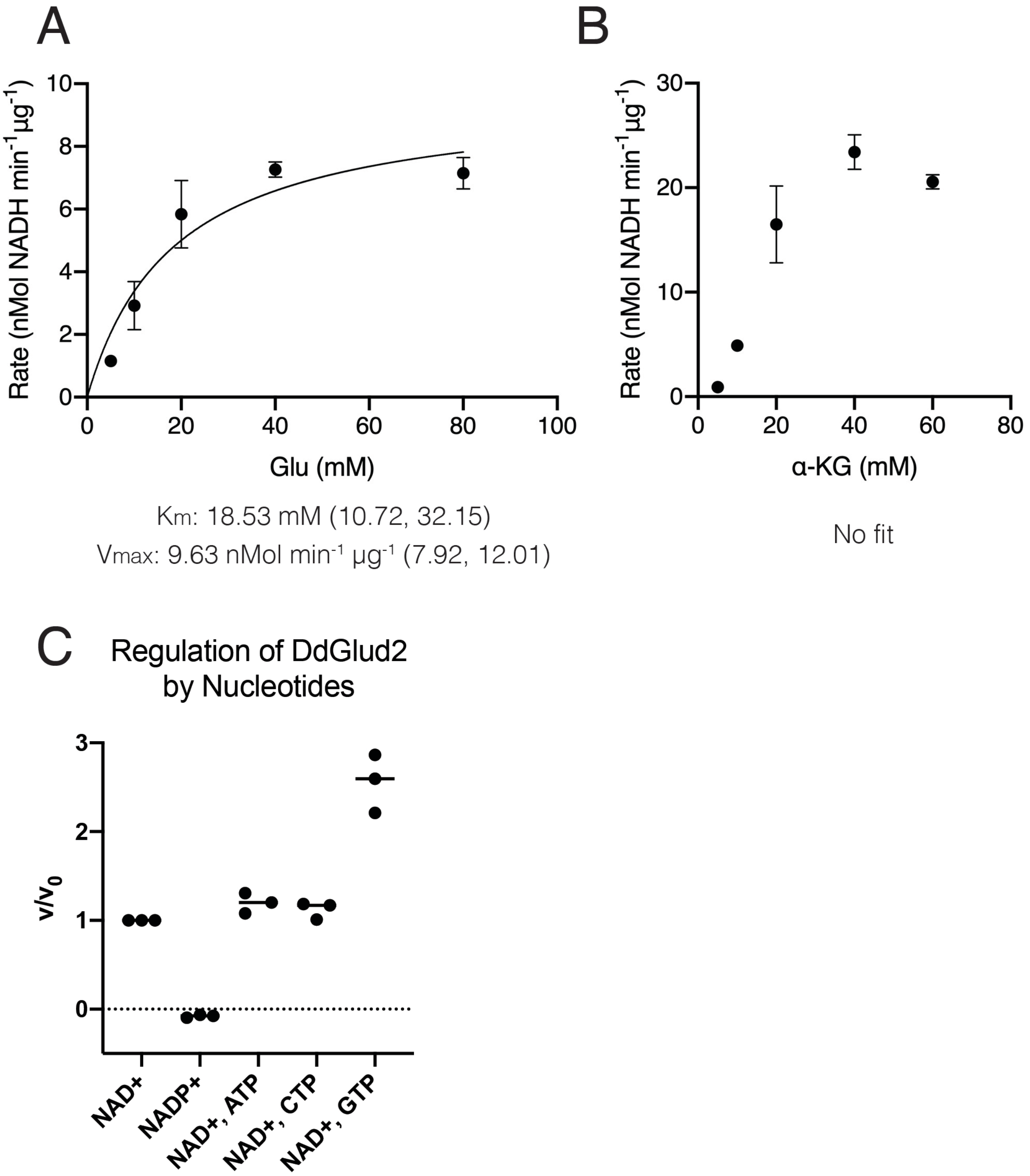

Figure S8 Kinetic properties of *Dd*Glud2. (A) Rate plot of *Dd*Glud2 in the presence of excess NAD+ and varying concentrations of glutamic acid. Rate was determined by spectrophotometric measurement of NAD+ reduction to NADH at 340nm. Km and Vmax, calculated by nonlinear regression fitting to Michaelis-Menten kinetics, are indicated below the plot with a 95% confidence interval. **(B)** Rate plot of *Dd*Glud2 in the presence of excess NADH and varying concentrations of α-KG. Rate was determined as in (A). **(C)** Comparison of *Dd*Glud2 reaction rate using NAD+ or NADP as a cofactor, or with 1mM ATP, CTP, and GTP present in the reaction. Reactions in (C) were performed with 10 mM Glu and 1 mM NAD+. The ratio v/v0 expresses the rate of the experimental reaction divided by rate in the presence of NAD+ and no nucleotide (v0.)

**Gene symbol (Gomez-Valero)**	**Gene symbol (Burstein)**	**% sequence identity vs Lart1**	**BLAST alignment length**	**species (strain)**
lpg0181	lpg0181	100	303	*Legionella pneumophila* Philadelphia
lpp0245	-	86	303	*Legionella pneumophila* Paris
lpl0244	-	85	303	*Legionella pneumophila* Lens
LpFRAB2158	-	85	304	*Legionella pneumophila* subsp. fraseri Dallas *1E*
LboA2942	Lboz_3623	76	303	*Legionella bozemanii (Fluoribacter bozemanae)*
Ltuc0973	Ltuc_1293	74	303	*Legionella tucsonensis*
LanA3764	Lani_2348	70	303	*Legionella anisa*
Lstei2883	Lstg_1741	70	303	*Legionella steigerwaltii*
-	Lste_0412	70	303	*Legionella steelei*
Lche0916	Lche_2404	64	303	*Legionella cherrii*
LduN231416	Ldum_0918	61	302	*Legionella (Fluoribacter) dumoffii*
Lsan4051	Lsan_1903	61	303	*Legionella santicrucis*
Lci2159	Lcin_2245	59	303	*Legionella cincinnatiensis*
Lno2069	-	59	303	*Legionella norrlandica*
llo2453	LLO_2453	58	303	*Legionella longbeachae*
LgoB0260	Lgor_1579	57	303	*Legionella (Fluoribacter) gormanii*
Lsain2178	Lsai_0094	57	303	*Legionella sainthelensi*
Lwad0791	-	55	303	*Legionella wadsworthii*
Lwal2311	Lwal_1589	48	303	*Legionella waltersii*
Lspi0543	Lspi_0096	42	304	*Legionella spiritensis*
Llan1731	Llan_2583	41	304	*Legionella lansingensis*
LmasA2084	-	25	132	*Legionella massilensis*
Lspi0719	Lspi_0863	23	192	*Legionella spiritensis*
Lis0071	Lisr_1114	22	283	*Legionella israelensis*
Lspi1868	Lspi_1980	21	215	*Legionella spiritensis*

Table S1: List of homologs of Lart1 (lpg0181) collected by BLAST search. For *L. pneumophila*, only selected strains shown. Note *Legionella spiritensis* has three Lart1 paralogs. Gene identifiers as per Gomez-Valero et al. (2018) – first column and as per Burstein et al. (2016) – second column.

Top Scoring proteins in Avidin IP - UNIQUE TO WT			
Protein Accession	Name	Score (WT)	Molecular Weight, Da
splP33327IDHE2	**NAD-specific glutamate dehydrogenase (GDH2)**	181	**124,254**
splQ03690ICLU	**Clustered mitochondria protein 1**	81	145,076
splQ3E764ITMA7	**Translation machinery-associated protein 7**	71	6,937
splP31539IHS104	**Heat shock protein 104 O**	64	101,972
splP08067IUCRI	**Cytochrome b-c1 complex subunit Rieske, mitochondrial**	63	23,350

Table S2: Curated list of proteins enriched from yeast lysate by streptavidin agarose following incubation with WT Lart1, Lart1 E137A, and biotin-17-NAD+. Proteins were identified by mass spectrometry. This table includes only proteins unique to the Lart1 WT-treated samples. Proteins are listed along with their MASCOT protein score and theoretical molecular weight.

Enzyme	Substrate	K_{cat} (min^{-1})	K_m (μM)	K_{cat}/K_m ($min^{-1}\ \mu M$)
Lart1	DdGlud2	0.15	2.2	0.68
C3larvin toxin (C3 family) from *P. larvae* (34)	RhoA-GST	5.26	16.8	0.31
iota$_a$ toxin from *C. perfringes* (36)	Actin	441	$1.5x10^{-4}$	$2.9x10^{6}$
Scabin toxin (Pierisin family) from *S. scabies* (35)	dG	14	302	0.46
ExoT toxin from *P. aeruginosa* (37)	GST-Crk-I	154	16	9.63

Table S3: Enzyme kinetics of selected mono-ADP-Ribosyltransferases. Reported kinetic parameters for bacterial mono-ARTs related to Lart1 were obtained from the literature. All parameters were obtained with respect to the protein or nucleoside substrate. dG: 2'-deoxyguanosine.
